# Structural basis for the blockade of MATE multidrug efflux pumps

**DOI:** 10.1038/ncomms8995

**Published:** 2015-08-06

**Authors:** Martha Radchenko, Jindrich Symersky, Rongxin Nie, Min Lu

**Affiliations:** 1 Department of Biochemistry and Molecular Biology, Rosalind Franklin University of Medicine and Science, 3333 Green Bay Road, North Chicago, Illinois 60064, USA

## Abstract

Multidrug and toxic compound extrusion (MATE) transporters underpin multidrug resistance by using the H^+^ or Na^+^ electrochemical gradient to extrude different drugs across cell membranes. MATE transporters can be further parsed into the DinF, NorM and eukaryotic subfamilies based on their amino-acid sequence similarity. Here we report the 3.0 Å resolution X-ray structures of a protonation-mimetic mutant of an H^+^-coupled DinF transporter, as well as of an H^+^-coupled DinF and a Na^+^-coupled NorM transporters in complexes with verapamil, a small-molecule pharmaceutical that inhibits MATE-mediated multidrug extrusion. Combining structure-inspired mutational and functional studies, we confirm the biological relevance of our crystal structures, reveal the mechanistic differences among MATE transporters, and suggest how verapamil inhibits MATE-mediated multidrug efflux. Our findings offer insights into how MATE transporters extrude chemically and structurally dissimilar drugs and could inform the design of new strategies for tackling multidrug resistance.

## Abstract (web summary)

MATE transporters confer bacterial multidrug resistance by transporting drugs across cell membranes. Here, the authors present new structures of the DinF and NorM MATE transporters and reveal insights into the mechanisms of substrate transport and MATE inhibition by small-molecule pharmaceuticals.

## Graphical abstract

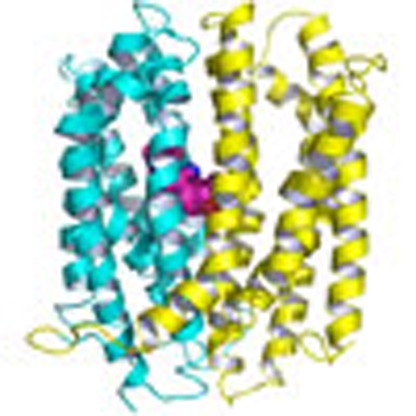

The inexorable rise in multidrug resistance outpaces the tempo of drug discovery and heralds a public-health crisis^1^,^2^. Integral membrane proteins belonging to the multidrug and toxic compound extrusion (MATE) family contribute to multidrug resistance by using either the Na^+^ or H^+^ electrochemical gradient to extrude drugs across cell membranes^3^,^4^,^5^. MATE proteins identified thus far can be separated into the NorM, DinF and eukaryotic subfamilies based on amino-acid sequence similarity^3^. The past 5 years have witnessed tremendous strides made in elucidating the molecular structures of MATE transporters. To date, the X-ray structures of Na^+^-dependent NorM transporters from *Vibrio cholerae* (NorM-VC)^6^ and *Neisseria gonorrhoeae* (NorM-NG)^7^, as well as H^+^-dependent DinF transporters from *Pyrococcus furiosus* (PfMATE)^8^ and *Bacillus halodurans* (DinF-BH)^9^ have been reported.

Those crystal structures revealed a similar protein fold comprising 12 membrane-spanning segments (TM1-TM12), they nevertheless suggested different arrangement of cation- and substrate-binding sites between the NorM and DinF proteins^8^,^9^, hinting at substantial mechanistic diversity among the MATE transporters^9^. Despite such progress, great uncertainties persist in our understanding of how MATE transporters expel drugs and more importantly, how they can be counteracted. In particular, although the structures of PfMATE bound to macrocyclic-peptide inhibitors had been revealed^8^, their mechanistic interpretation is far from straightforward, given the controversy surrounding the proposed antiport mechanisms^9^,^10^,^11^,^12^.

On one side sit the PfMATE structures determined at high (7.0–8.0) and low (6.0–6.5) pH, where TM1 is more bent in the latter, leading to suggestions that the protonation of PfMATE^D41^ triggers the bending of TM1 to extrude drugs^8^. This indirect coupling mechanism assumes that PfMATE^D41^ is deprotonated at pH ∼7 but protonated at pH ∼6. In the published structures, however, PfMATE^D41^ has a calculated pKa of <4 and is likely deprotonated in the low-pH structure^9^. On the other side are the DinF-BH structures determined in the presence and absence of a bound substrate^9^, consistent with a direct-competition-based mechanism wherein H^+^ and substrate compete for DinF-BH^D40^ (equivalent of PfMATE^D41^). Furthermore, the calculated and experimentally determined pKa values for DinF-BH^D40^ converged to ∼7, qualifying DinF-BH^D40^ as a physiologically relevant protonation site^9^,^13^,^14^. Although this direct coupling mechanism would not require substantial protein conformational changes elicited by protonation, including TM1 bending, this scenario does not exclude the possibility of such changes.

In this work, we set out to determine the X-ray structure of a protonation-mimetic mutant of DinF-BH. This structure reveals an H-bonding network situated directly adjacent to the multidrug-binding site in DinF-BH and supports a direct-competition-based antiport mechanism^9^. Furthermore, we determined the crystal structures of DinF-BH and NorM-NG in complexes with verapamil, a broad-spectrum inhibitor of multidrug efflux pumps. In the co-crystal structures, verapamil preoccupies the multidrug-binding sites in DinF-BH and NorM-NG by adopting surprisingly different conformations, deterring drugs from binding the MATE antiporters for extrusion. Combining crystallographic and biochemical studies, we shed new light on the mechanistic features that allow MATE transporters to extrude chemically and structurally different drugs, as well as the mechanism whereby verapamil inhibits mechanistically distinct DinF and NorM transporters.

## Results

### Structure of a protonation-mimetic mutant of DinF-BH

**Figure 1:**
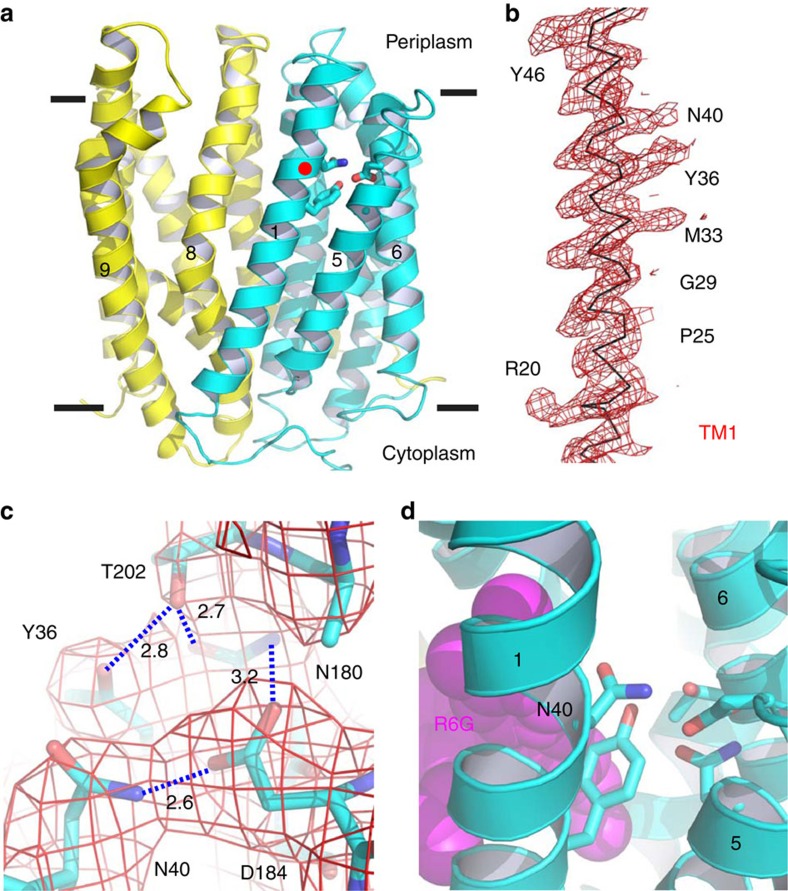
Structure of DinF-BH^D40N^. (**a**) Structure of DinF-BH^D40N^ as viewed parallel to the membrane. The N (residues 3–227) and C (residues 228–448) domains are coloured cyan and yellow, respectively. Relevant transmembrane helices are numbered and amino acids are drawn as sticks. Red dot highlights N40. (**b**) Experimental electron density for TM1. The map (red mesh) was calculated to 3.0 Å resolution and contoured at 1.5 σ, using density-modified MIRAS phases. Relevant amino-acid side chains are numbered. (**c**) Experimental electron density for the H-bond network. The map (red wire) was calculated to 3.0 Å resolution and contoured at 1.5 σ, using density-modified MIRAS phases. Relevant amino-acid side chains are drawn as sticks. H-bonds are indicated by dashed lines and the distances (in angstroms) are indicated. (**d**) Close-up view of amino acids (sticks) that form the H-bond network, with N40 numbered. The substrate-binding site is indicated by R6G (magenta spheres), which was taken from the R6G-bound structure^9^.

We first explored the possibility of protonation-induced TM1 bending in DinF-BH by seeking the structure of a protonated state. We replaced DinF-BH^D40^ with asparagine, mimicking a constitutively protonated aspartate^9^. This mutation abrogated the transport function^9^, likely because the protein was trapped in a protonated state. We determined the structure of DinF-BH^D40N^ to 3.5 Å resolution by using molecular replacement and multiple isomorphous replacement and anomalous scattering (MIRAS) phasing (Fig. 1, Supplementary Table 1). We also soaked DinF-BH^D40N^ crystals into low pH (∼4) solutions, and determined the structure to 3.0 Å resolution by combining molecular replacement and MIRAS phasing. The low pH-soaked structure of DinF-BH^D40N^ is largely identical to the un-soaked one (root mean squared deviation (r.m.s.d.) <0.5 Å for 447 Cα positions), ascertaining that the protein was indeed locked into a protonated state.

Overall, the structure of DinF-BH^D40N^ resembles that of the extracellular-facing, substrate-bound DinF-BH (ref. 9), with an r.m.s.d. of <1 Å over 447 Cα positions (Fig. 1a, Supplementary Fig. 1). Tellingly, TM1 in DinF-BH^D40N^ is no more bent than that of DinF-BH, suggesting the notion that TM1 is kinked in an extracellular-facing, protonated MATE transporter^8^ is not always applicable (Fig. 1b). Significantly, TM1 in DinF-BH^D40N^ is not involved in any crystal packing interactions, indicating that its straight conformation is unlikely an idiosyncratic crystallization artifact (Supplementary Fig. 2). Furthermore, DinF-BH^D40^ is replaced by a non-protonable serine in the H^+^-coupled human MATE transporters hMATE1 and hMATE2 (refs 15, 16), implying that a protonated and bent TM1 is unlikely a common feature of H^+^-dependent MATE proteins (Supplementary Fig. 3).

### An H-bonding network involving DinF-BH^D40N^

Aside from the similarities, the DinF-BH^D40N^ structure does bear several differences from the deprotonated DinF-BH (ref. 9). DinF-BH^D40N^ and DinF-BH crystallized in the same crystal forms with similar unit-cell dimensions, suggesting that the differences between those two structures are caused by the mutation and low pH rather than by different crystal packing environments. The most functionally important difference involves the side chain of DinF-BH^D40N^, which participates in an H-bonding network also involving DinF-BH^Y36^ (TM1), DinF-BH^N180^, DinF-BH^D184^ (TM5) and DinF-BH^T202^ (TM6; Fig. 1c). In this network, the side chain of DinF-BH^D40N^ forms an H-bond with that of DinF-BH^D184^. By contrast, in the deprotonated DinF-BH^9^, no such interaction was observed between DinF-BH^D40^ and DinF-BH^D184^ (Supplementary Fig. 4).

Furthermore, DinF-BH^D40^ appears to make a close-range charge–charge interaction with a cationic substrate within a voluminous, membrane-embedded multidrug-binding chamber^9^ (Supplementary Fig. 4). Notably, neutralizing DinF-BH^D40^ drastically reduced the substrate-binding affinity of DinF-BH^9^. Taken together, we suggest that H^+^ evokes substrate release by breaking the charge–charge interaction between the deprotonated DinF-BH^D40^ and the bound substrate, and by engaging the protonated DinF-BH^D40^ in an H-bonding interaction with DinF-BH^D184^.

Our crystallographic results also implicated DinF-BH^Y36^ (TM1), DinF-BH^N180^, DinF-BH^D184^ (TM5) and DinF-BH^T202^ (TM6) in multidrug efflux, as they form an H-bonding network with DinF-BH^D40N^. To probe their functional importance, we replaced those amino acids individually with alanine or asparagine (in case of DinF-BH^D184^), and found that these mutants are expressed at similar levels to that of DinF-BH (Supplementary Fig. 5). We had previously discovered that DinF-BH^Y36^ was not essential for the transport function^9^, we thus examined the untested mutants by using the drug-resistance assay^7^,^9^. We observed that DinF-BH^N180A^, DinF-BH^D184A^, DinF-BH^D184N^ and DinF-BH^T202A^ all exhibited severely impaired transport function, since they could no longer confer cellular resistance against drugs (Supplementary Fig. 4). Consistent with these observations, those mutations abolished the ability of DinF-BH to catalyse drug/H^+^ exchange in everted membrane vesicles^9^. Although the mutation of DinF-BH^Y36^ failed to abrogate the transport function, it altered the ability of DinF-BH to extrude drugs (see below).

Of particular interest, the deprotonation of DinF-BH^D184^ appeared to be essential for multidrug extrusion, as a D184N substitution neutralized DinF-BH^D184^ and abolished the transport function (Supplementary Fig. 4). Despite such functional importance, DinF-BH^D184^ is substituted by a non-protonatable asparagine in members of the NorM and eukaryotic subfamilies (Supplementary Fig. 3), some of which are known to function as H^+^-coupled transporters^15^,^16^,^17^,^18^,^19^. Furthermore, prior studies had demonstrated that the mutation of NorM-VC^N174^ (equivalent of DinF-BH^N180^) had little effect on the transport function^11^. These findings thus implied that eukaryotic MATE and NorM transporters have evolved different transport mechanisms from that of DinF-BH.

### Inhibition of multidrug extrusion by verapamil

**Figure 2:**
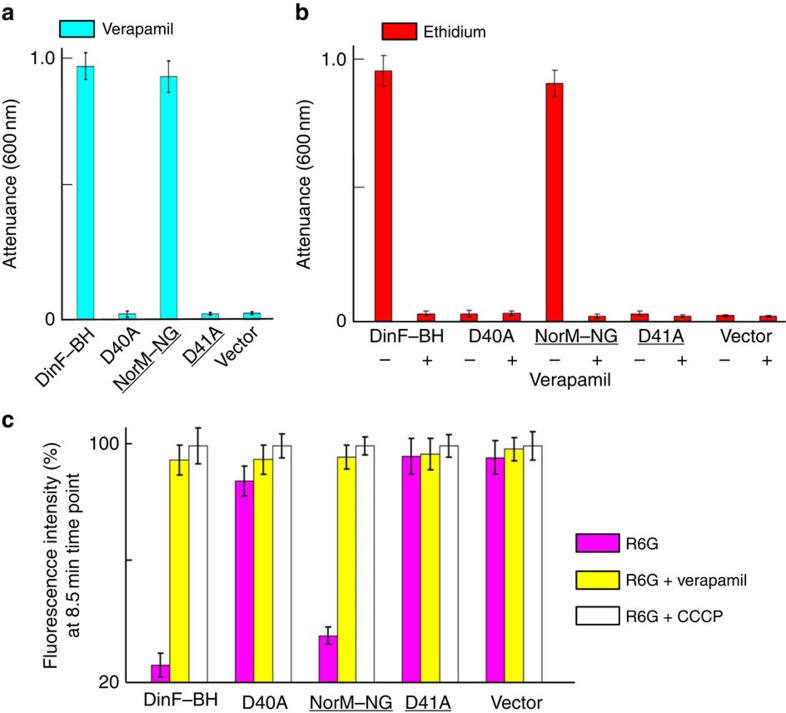
Inhibition of multidrug efflux by verapamil. (**a**,**b**) The inhibition of bacterial growth as monitored by attenuance at 600 nm. The bacteria expressing the MATE variants or pET-15b were grown in the presence of 150 μM (∼74 μg ml^−1^) verapamil (**a**); or in the presence of 0.5 μg ml^−1^ of ethidium, with or without 50 μM verapamil (**b**). (**c**) R6G accumulation in bacteria expressing the MATE variants or pET-15b in the absence (magenta) or presence of 100 μM verapamil (yellow), or 25 μM CCCP (white), as examined by the measurement of R6G fluorescence. Error bars indicate s.d. among three biological replicates.

We next investigated how DinF-BH and NorM-NG can be counteracted by small-molecule (Mr <500) inhibitors^20^,^21^. We focused on verapamil, a currently marketed ion-channel blocker as well as a broad-spectrum inhibitor of multidrug transporters^22^,^23^,^24^, including hMATE1 and hMATE2 (refs 15, 25). We observed that verapamil is moderately toxic to bacteria (minimal inhibitory concentration ∼150 μM), and the expression of DinF-BH or NorM-NG, not the inactive MATE mutants^7^,^9^, could protect the bacteria from the toxicity of verapamil (Fig. 2a).

Moreover, verapamil markedly reduced the ability of DinF-BH or NorM-NG to impart cellular resistance toward cytotoxic compounds, presumably by blocking the MATE-mediated drug efflux (Fig. 2b). Consistent with its inhibitory effects on drug efflux, verapamil drastically enhanced the accumulation of cytotoxic drugs within the bacteria expressing DinF-BH or NorM-NG, but had little or no effect on the cells harbouring the inactive MATE mutants^7^,^9^ (Fig. 2c).

DinF-BH or NorM-NG could confer cellular resistance to verapamil (Fig. 2a), implying that verapamil can be extruded by DinF-BH or NorM-NG, and that verapamil shares a similar binding site and transport pathway with known MATE substrates including rhodamine 6G (R6G). Indeed, verapamil reduced the binding of radiolabeled substrate ([^3^H]-R6G) to DinF-BH or NorM-NG *in vitro*, although less effectively than other tested substrates (Supplementary Fig. 6), probably owing to its lower binding affinity. Altogether, our data suggested that verapamil exerts inhibitory effects on MATE-mediated multidrug efflux by interfering with drug binding.

### Structure of verapamil-bound DinF-BH

**Figure 3:**
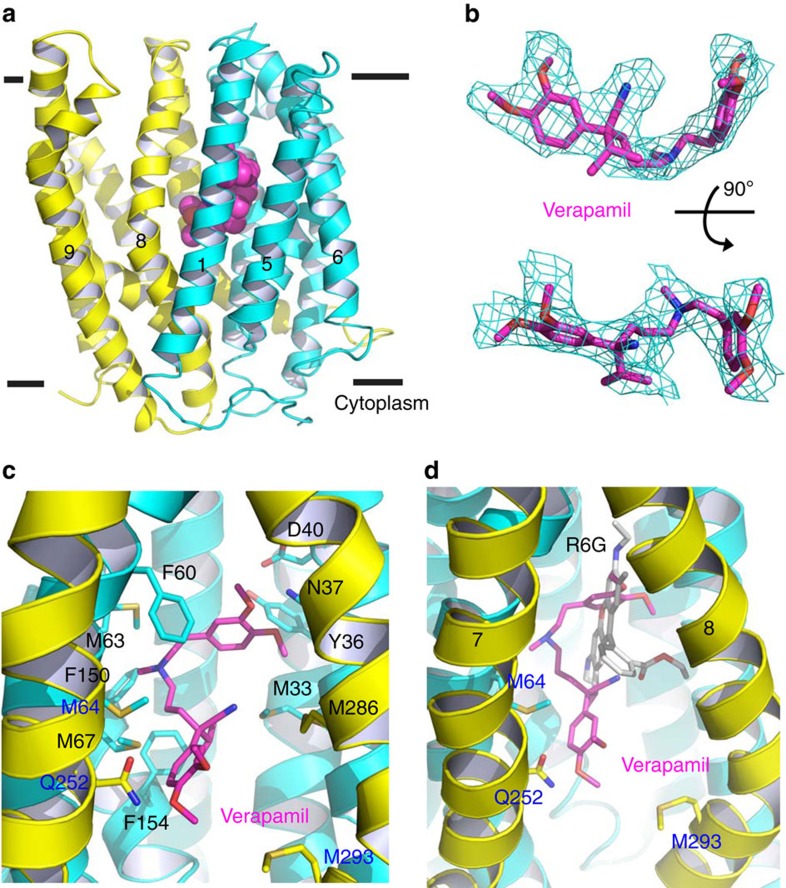
Structure of verapamil-bound DinF-BH. (**a**) DinF-BH is shown in ribbon rendition and coloured as in Fig. 1a, whereas the bound verapamil is drawn as magenta spheres. (**b**) Fitting of the asymmetric verapamil to the experimental electron density map (cyan mesh), which was calculated to 3.0 Å resolution using density-modified MIRAS phases and contoured at 1.5 σ. The map was overlaid onto the final model of verapamil (magenta sticks). Top view is related to the bottom one by a rotation of 90°. (**c**) Close-up view of the verapamil-binding site, verapamil (magenta) and relevant amino acids are displayed as stick models. (**d**) Comparison of verapamil- and R6G-binding sites, with verapamil (magenta), R6G (grey) and amino acids that only bind to verapamil drawn as stick models.

To reveal how verapamil blocks drug binding, we co-crystallized DinF-BH with verapamil, and determined the co-structure to 3.0 Å resolution by using molecular replacement and MIRAS phasing (Fig. 3a, Supplementary Table 2). The structure of verapamil-bound, extracellular-facing DinF-BH is very similar to that of the R6G-bound form^9^, with an r.m.s.d. of <0.5 Å for 447 Cα positions. Apparently, the structure of the ligand-binding chamber is not appreciably altered by the bound verapamil, whose orientation was clearly defined by the experimental electron density within the large, voluminous multidrug-binding chamber (Fig. 3b, Supplementary Fig. 7).

About half-way across the membrane bilayer, verapamil adopts a rather extended conformation and makes numerous close-range interactions with both the N (TM1-TM6) and C (TM7-TM12) domains of DinF-BH (Fig. 3c). Specifically, the side chains of N37 (TM1) and Q252 (TM7) form H-bonds with the bound verapamil, whereas those of M33, Y36 (TM1), F60, M63, M64, M67 (TM2), F150, F154 (TM4), M286 and M293 (TM8) make contacts through van der Waals interactions (Fig. 3c). The hydrophobic, methionine-rich chamber in DinF-BH is reminiscent of the protein-binding groove seen in the protein targeting factor Get3, which appears to afford Get3 the ability to bind a wide variety of hydrophobic amino-acid sequences^26^, implying a similar molecular design for ‘poly-specific’ protein–ligand interactions.

Moreover, although the positively charged verapamil^27^ is enclosed in a binding chamber with an overall negative surface electrostatic potential^9^, no close-range ionic interaction could be found between DinF-BH and verapamil (Fig. 3c). Structural comparison of the verapamil- and R6G-bound DinF-BH revealed overlapping, but not identical, binding sites for both ligands^9^ (Fig. 3d). Notably, all the verapamil-contacting amino acids except M64, Q252 and M293 make contacts with R6G. Therefore, the binding of verapamil and R6G to DinF-BH is mutually exclusive, offering a structural rationale for the obstruction of multidrug efflux by verapamil.

### Interactions between DinF-BH and different ligands

In the R6G-bound DinF-BH, D40 makes a close-range ionic contact with the cationic substrate^9^ (Supplementary Fig. 4). By contrast, in the verapamil-bound structure, D40 is positioned ∼9 Å away from atom N8 in verapamil, the ternary ammonium group carrying a positive charge^27^. Therefore, verapamil may interact with D40 through long-range electrostatic forces^28^,^29^,^30^, which is likely enabled by the hydrophobic, low dielectric environment within the membrane-embedded drug-binding chamber. Furthermore, unlike R6G, the two aromatic rings in verapamil linked through N8 are not conjugated. As a result, the positive charge in verapamil is localized on N8 rather than scattered over the aromatic rings as in R6G, further limiting the ability of verapamil to make favourable electrostatic interactions with D40. In addition, R6G inserts more deeply into the drug-binding chamber than verapamil does, making a larger number of H-bonding and van der Waals interactions with DinF-BH (Fig. 3d).

In addition, verapamil seems intrinsically more flexible than R6G, and the flexibility of verapamil is expected to be substantially reduced on its binding to DinF-BH. As such, the loss of conformational entropy due to DinF-BH binding is likely to be greater for verapamil than R6G. Since changes in conformational entropy can contribute significantly to the free energy of protein–ligand association^31^, the greater flexibility of verapamil may also contribute to the weaker interactions seen between DinF-BH and verapamil, as opposed to R6G.

Therefore, in comparison with verapamil, R6G makes a larger number of contacts with DinF-BH, including a close-range ionic interaction; and the conformational entropy of R6G is probably less reduced by its association with DinF-BH. These two differences help to explain why R6G competed more effectively against radioligand than verapamil for DinF-BH binding (Supplementary Fig. 6), but they also prompt the question of whether verapamil, as other known substrates of DinF-BH, is extruded via the H^+^-coupled, direct-competition-based antiport mechanism^9^. To address this question, we studied the DinF-BH-mediated verapamil transport by using a fluorescence-based, drug/H^+^ antiport assay^9^,^32^.

We observed that verapamil could trigger the export of H^+^ in everted membrane vesicles containing DinF-BH, but not in those vesicles harbouring an inactive mutant, DinF-BH^D40A^ (Supplementary Fig. 8). This finding suggested that despite the lack of close-range ionic interaction between D40 and verapamil seen in the co-structure (Fig. 3c), the transport of verapamil by DinF-BH remains coupled to the D40-dependent, counter-movement of H^+^ across the cell membrane. Therefore, these data implied that DinF-BH catalyses verapamil/H^+^ exchange and in the extracellular-facing transporter, protonation triggers the export of verapamil by disrupting the aforementioned, long-range electrostatic interaction between D40 and verapamil.

### Mutational effects on the verapamil-binding site in DinF-BH

**Figure 4:**
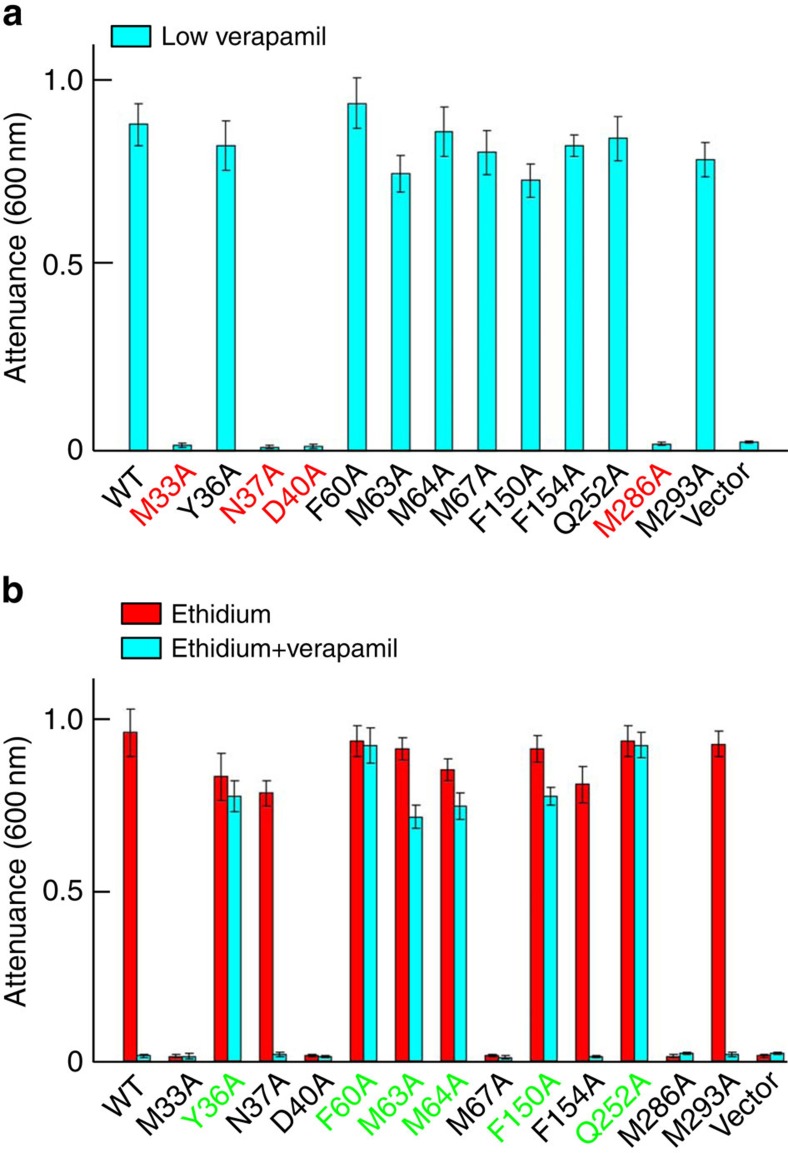
Functional importance of the verapamil-binding site in DinF-BH. The inhibition of bacterial growth as measured by attenuance at 600 nm. Bacteria expressing the DinF-BH variants or pET-15b were grown in the presence of 150 μM verapamil (cyan column, **a**); or in the presence of 0.5 μg ml^−1^ ethidium, with (cyan column) or without (red column) 50 μM verapamil (**b**). Error bars indicate s.d. among three biological replicates.

To corroborate the biological relevance of our crystallographic results, we replaced verapamil-contacting amino acids individually by alanines. Although those mutations had no measurable effects on DinF-BH expression (Supplementary Fig. 5), mutation of M33, N37, D40 or M286 abolished the ability of DinF-BH to impart cellular resistance to verapamil (Fig. 4a). By contrast, mutation of Y36, F60, M63, M64, M67, F150, F154, Q252 or M293 had little deleterious effect. These observations implied that M33, N37, D40 and M286 play critical roles in transporting verapamil, while the other amino acids are non-essential. Notably, previous studies^9^ indicated that M33, D40, M67 and M286 play pivotal roles in extruding drugs, whereas N37 does not (Fig. 4b). Collectively, these results suggested that verapamil and other known substrates share a similar but not identical transport pathway, involving M33, D40 and M286.

**Figure 5:**
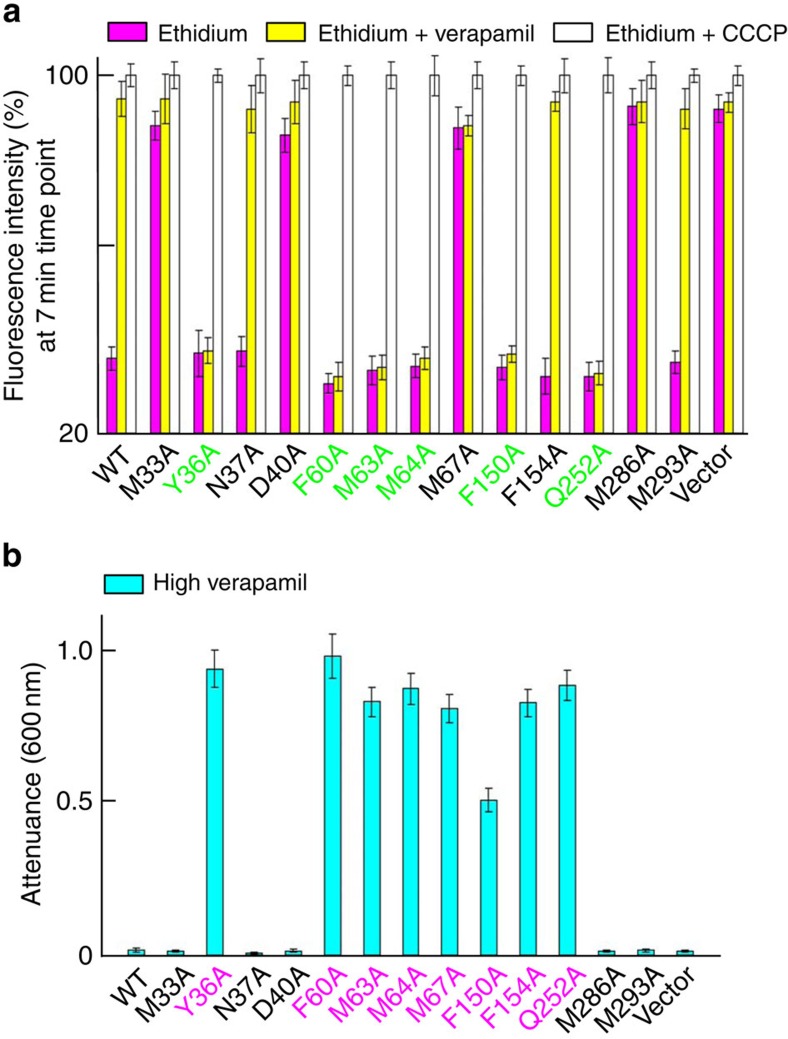
Mutational effects on the verapamil-binding site in DinF-BH. (**a**) Fluorescence-based measurement of the accumulation of ethidium in bacteria expressing the DinF-BH variants or pET-15b, in the absence (magenta) or presence of 100 μM verapamil (yellow), or 25 μM CCCP (white). (**b**) The inhibition of bacterial growth as measured by attenuance at 600 nm. Bacteria expressing the indicated DinF-BH variants or pET-15b were grown in the presence of 300 μM verapamil. Error bars indicate s.d. among three biological replicates.

Moreover, mutations of Y36, F60, M63, M64, F150 and Q252 rendered DinF-BH-mediated drug efflux resistant to verapamil treatment. Specifically, verapamil could no longer suppress the ability of those mutants to confer cellular resistance to drugs (Fig. 4b), nor could verapamil enhance the accumulation of drugs in bacteria expressing those mutants (Fig. 5a). It is likely that, although those mutations failed to abrogate the transport function, they led to the loss of some close-range interactions between verapamil and DinF-BH, as seen in the co-structure (Fig. 3c), thereby reducing the sensitivity of drug efflux towards verapamil inhibition.

Furthermore, the alanine substitution of Y36, F60, M63, M64, M67, F150, F154 or Q252 enabled DinF-BH to confer enhanced cellular resistance to verapamil (Fig. 5b). This observation implied that those mutations altered the interactions between DinF-BH and verapamil (Fig. 3c) to such a degree that DinF-BH could transport verapamil more effectively. It seems conceivable that, although those mutations weakened verapamil binding by disrupting the close-range interactions seen between DinF-BH and verapamil (Fig. 3c), they more than offset the adverse effects on the transport by facilitating the conformational changes that DinF-BH undergoes during verapamil extrusion, and/or promoting the release of verapamil during export.

Although plausible, this interpretation merits caution, as our data shed little light on whether or how those mutations affect the conformational changes in DinF-BH during multidrug transport. Notwithstanding this limitation, our findings supported the functional relevance of the co-crystal structure, suggesting that DinF-BH extrudes different substrates via slightly different transport routes, and implying that Y36, F60, M63, M64, F150 and Q252 play regulatory roles in the transport function, for example, by affecting protein conformational changes. The overlapping but non-identical transport paths and ligand-binding sites in DinF-BH appear to underlie its functional versatility of extruding chemically and structurally distinct compounds, including R6G and verapamil.

### Structure of verapamil-bound NorM-NG

**Figure 6:**
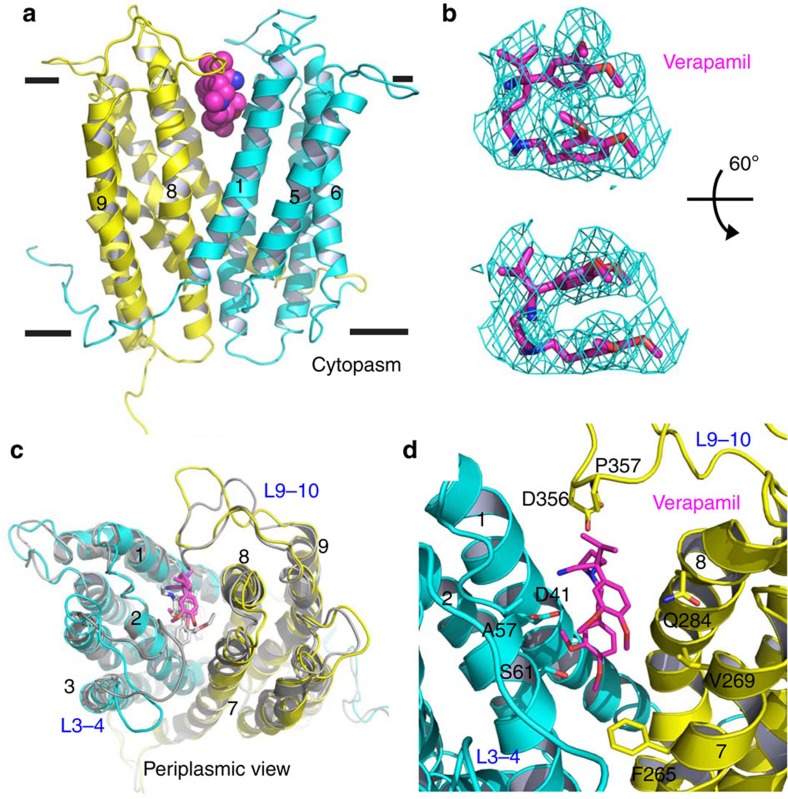
Structure of verapamil-bound NorM-NG. (**a**) Structure of verapamil-bound NorM-NG as viewed from the membrane plane, with the bound verapamil drawn as magenta spheres. The N (residues 5–230) and C (residues 231–459) domains are in cyan and yellow, respectively. (**b**) Fitting of the asymmetric verapamil to the experimental electron density map (cyan wire), which was calculated to 3.0 Å resolution using density-modified MIRAS phases and contoured at 2.0 σ. The map was overlaid onto the final model of verapamil (magenta sticks). Top view is related to the bottom one by a rotation of 60°. (**c**) Structural overlay of verapamil- and R6G-bound NorM-NG, as viewed from the periplasmic side. The verapamil-bound NorM-NG is coloured as in (**a**) while the R6G-bound transporter is in grey. Verapamil (magenta) and R6G (grey) are in stick representation. (**d**) Close-up view of verapamil-binding site, with verapamil (magenta) and relevant amino acids drawn as sticks.

Hypothesizing that NorM-NG would interact with verapamil differently from DinF-BH, we determined the structure of verapamil-bound NorM-NG to 3.0 Å resolution by combining molecular replacement and MIRAS phasing (Fig. 6a, Supplementary Table 3). The verapamil-binding site in extracellular-facing NorM-NG was located from experimental electron density that is consistent with both the size and shape of the bound ligand (Fig. 6b, Supplementary Fig. 9).

Notably, the verapamil-binding site overlaps substantially with the multidrug-binding site in NorM-NG^7^, with verapamil and drugs including R6G following similar trajectories within the central cavity and projecting themselves towards both the N and C domains (Fig. 6c). Furthermore, the bound verapamil in NorM-NG adopts a folded conformation, with both aromatic rings stacked on top of each other. This double-layered conformation bore little resemblance to that seen in verapamil-bound DinF-BH, which does not involve π-stacking between the two aromatic rings (Fig. 3b).

Although verapamil binds in a similar location to that of R6G, substantial structural differences were found between the verapamil- and R6G-bound NorM-NG, with r.m.s.d. exceeding 2 Å over 459 aligned Cα positions (Fig. 6c). Since the verapamil- and R6G-bound NorM-NG crystallized in the same crystal forms with similar unit-cell dimensions, those structural difference are likely due to the differences in the bound ligands. The most notable differences between the R6G- and verapamil-bound NorM-NG structures reside in two extracellular loops termed L3–4 and L9–10, which cap the multidrug-binding cavity in the transporter^7^. In verapamil-bound NorM-NG, both L3–4 and L9–10 moved away from the multidrug-binding cavity (Fig. 6c, Supplementary Fig. 10).

The largest movement was made by L3–4, to such an extent that L3–4 can no longer insert amino-acid side chains into the central cavity to interact with the bound verapamil (Fig. 6d). L9–10 also shifted away from the multidrug-binding cavity, interacting with verapamil only modestly. Overall, a small number of amino-acid side chains make direct contacts with the bound verapamil in NorM-NG. Among them, S61 (TM2), Q284 (TM8) interact with verapamil through H-bonding interactions, while A57 (TM2), F265, V269 (TM7) and P357 (L9–10) bind the ligand via van der Waals interactions (Fig. 6d). In addition, the carboxylate groups of D41 (TM1) and D356 (L9–10) are ∼6 and 3.5 Å away from N8 in verapamil, respectively.

This arrangement implies rather moderate electrostatic attraction between NorM-NG and verapamil, contrasting the numerous charge–charge interactions seen in the R6G-bound structure^7^. Apparently, although verapamil folds itself drastically to fit into the multidrug-binding cavity, it fails to make as many favourable interactions with NorM-NG as R6G does. Furthermore, for verapamil, the greater loss of conformational entropy on binding to NorM-NG, may also weaken its association with NorM-NG, as compared with R6G. Therefore, both the enthalpic and entropic factors may contribute to the poor ability of verapamil to compete against radioligand for NorM-NG binding *in vitro* (Supplementary Fig. 6).

### Importance of the verapamil-binding site in NorM-NG

**Figure 7:**
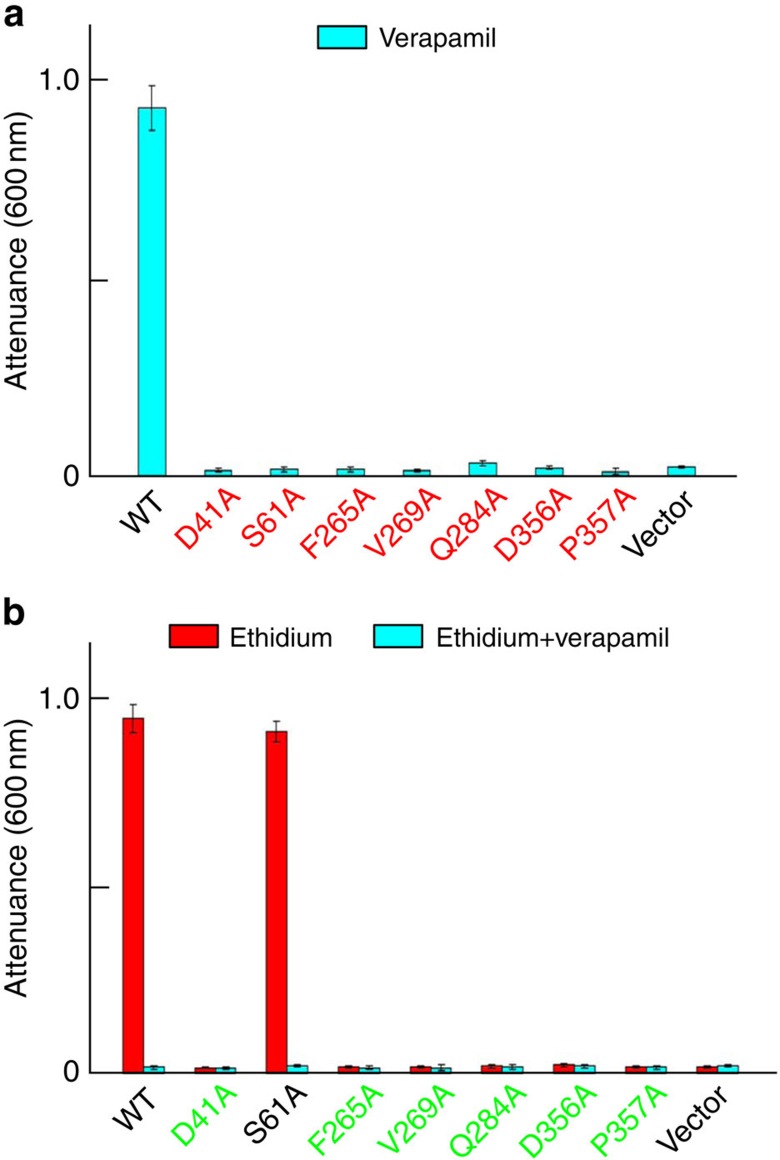
Biological importance of the verapamil-binding site in NorM-NG. The inhibition of bacterial growth as measured by attenuance at 600 nm. Bacteria expressing the NorM-NG variants or pET-15b were grown in the presence of 150 μM verapamil (cyan column, **a**); or in the presence of 0.5 μg ml^−1^ ethidium, with (cyan column) or without (red column) 50 μM verapamil (**b**). Error bars indicate s.d. among three biological replicates.

To affirm the observed interactions between NorM-NG and verapamil are of biological importance, we substituted the verapamil-binding amino acids individually by alanines. We observed that mutants D41A, S61A, F265L, V269A, Q284A, D356A and P357A were expressed at similar levels to that of NorM-NG (Supplementary Fig. 5), but they could no longer confer cellular resistance against verapamil (Fig. 7a), suggesting that those amino acids are essential for transporting verapamil. Notably, our prior studies^7^ had showed that the mutations of D41, F265, Q284 and D356 abolished the drug transport function, whereas mutation of S61 had little detrimental effect.

These data implied that verapamil and other known substrates (ethidium and R6G) share a similar but not identical transport route in NorM-NG, likely involving D41, F265, V269, Q284, D356 and P357. Furthermore, none of the tested mutations enabled NorM-NG to confer drug resistance in the presence of verapamil (Fig. 7b), contrasting what was observed for DinF-BH (Fig. 4b) and underscoring their mechanistic differences^9^. Despite such differences, both NorM-NG and DinF-BH appear to use slightly different subsets of amino acids for transporting chemically and structurally distinct drugs, which may represent a common theme for the ‘poly-specific’ MATE transporters^4^,^5^.

## Discussion

Our results suggested that the protonation of TM1 in DinF-BH induces the release of substrate without entailing significant structural changes in the extracellular-facing transporter, including the bending of TM1. This mechanistic feature puts DinF-BH in sharp contrast to NorM-NG, in which cation binding triggers substrate unbinding likely by shifting several transmembrane helices^7^. Furthermore, despite the substantial protein sequence similarity between DinF-BH and PfMATE (Supplementary Fig. 3), we found no structural proof for protonation-induced TM1-bending in the extracellular-facing DinF-BH. It may be relevant to note that DinF-BH was crystallized in the absence of exogenous lipids, whereas PfMATE was crystallized in the presence of monoolein, an exogenous lipid^8^. Moreover, the interactions between monoolein and PfMATE seen in the high-pH structure appeared to be different from those in the low-pH form^8^. This observation thus raises the possibility that the conformation of TM1 in PfMATE was affected by the pH-dependent interactions between monoolein and the transporter.

Furthermore, our data revealed remarkable mechanistic differences among the MATE transporters. Aside from the structural and functional differences between DinF-BH and NorM-NG, we uncovered previously undescribed mechanistic differences between DinF-BH and eukaryotic MATE transporters. On the basis of our crystallographic and biochemical data as well as protein sequence analysis, we suggest that eukaryotic MATE proteins including hMATE1 transport substrates via a different mechanism from that of DinF-BH. Given the amino-acid sequence similarity between hMATE1 and NorM-NG (Supplementary Fig. 3), and available biochemical data^7^,^15^, we argue that the H^+^-dependent hMATE1 is mechanistically more similar to Na^+^-coupled NorM-NG than to H^+^-coupled DinF-BH. As such, the structure of verapamil-bound NorM-NG rather than that of DinF-BH may serve as a useful starting point for examining the mechanistic interactions between verapamil and human MATE transporters.

Finally, the mechanistic differences between DinF-BH and NorM-NG are highlighted in their modes of interaction with verapamil. First, verapamil adopts different conformations to fit into the multidrug-binding site, which differs both in volume and chemical environment between DinF-BH and NorM-NG^7^,^9^. Second, while the overall structure of DinF-BH remains largely unaltered when the bound R6G is replaced by verapamil, NorM-NG undergoes pronounced conformational changes in two of its extracellular loops. These differences notwithstanding, in both DinF-BH and NorM-NG, verapamil precludes drugs from binding the transporter by preoccupying the substrate-binding site, which is also enabled by the structural flexibility of verapamil. Furthermore, our findings identified functionally important amino acids in DinF-BH and NorM-NG, many of which make direct contacts with verapamil in the co-structures. Our studies thus implicated verapamil as a potentially useful probe to uncover the therapeutically vulnerable sites on MATE transporters in general, opening new avenue of thought on battling multidrug resistance^33^.

## Methods

### Protein expression constructs

Full-length DinF-BH and NorM-NG were expressed with cleavable hexa- and deca-histidine tags at their C-termini, respectively^7^,^9^. Mutations were introduced into the *dinF-BH* and *norM-NG* genes by using the QuikChange method (Agilent Technologies) and were confirmed by DNA sequencing^7^,^9^. *Escherichia coli s*BL21 (DE3) Δ*acrAB*Δ*macAB*Δ*yojHI* cells^34^ were transformed with pET-15b vector containing the inserted genes encoding the DinF-BH and NorM-NG mutants.

The membrane expression levels of DinF-BH and NorM-NG were not affected by the mutations described in this study, as judged by western blot using an antibody against the His-tag (Supplementary Fig. 5). For the western blot, the antibody (Qiagen #34460) was diluted 2,500-fold before being mixed with the transfer membranes, and each sample examined on the gel was derived from cell membranes isolated from 80 μg *E. coli* BL21 (DE3) Δ*acrAB*Δ*macAB*Δ*yojHI* cells expressing the MATE variants.

### Protein expression and purification

DinF-BH^D40N^ was expressed and purified in the same manner as DinF-BH^9^. DinF-BH and NorM-NG were expressed and purified as follows. Briefly, *E. coli* BL21 (DE3) cells transformed with membrane protein expression vectors were grown in Luria–Bertani (LB) media to an attenuance of 0.5 at 600 nm and induced with 0.5 mM isopropyl β-D-1-thiogalactopyranoside (IPTG) at 37 °C for 3 h. Cells were collected by centrifugation and ruptured by multiple passages through a pre-cooled microfluidizer. All the membrane protein purification experiments were conducted at 4 °C.

Membranes were collected by ultracentrifugation and extracted with 1% (w/v) n-dodecyl-β-maltoside (DDM, Anatrace) in 20 mM HEPES-NaOH pH 7.5, 100 mM NaCl, 20% (v/v) glycerol and 1 mM tris(2-carboxyethyl)phosphine (TCEP). The soluble fraction was loaded onto Ni-NTA resin in 20 mM Hepes-NaOH pH 7.5, 100 mM NaCl, 25% glycerol, 0.02% DDM and 1 mM TCEP. Protein was eluted using the same buffer supplemented with 500 mM imidazole. The protein sample was promptly desalted and incubated with thrombin overnight. After thrombin cleavage the protein sample was desalted and concentrated to ∼15 mg ml^−1^ before it was further purified by using gel filtration chromatography (Superdex 200) in 20 mM Hepes-NaOH pH 7.5, 100 mM NaCl, 15% glycerol, 0.02% DDM and 1 mM TCEP. For ligand-binding experiments, the thrombin cleavage step was omitted. The expression levels of DinF-BH and NorM-NG are low, which may explain why the expression of known MATE transporters confers only modest levels of cellular resistance against drugs^4^,^5^.

The monobody with an N-terminal deca-histidine tag was expressed and purified as follows. Briefly, *E. coli* BL21 (DE3) cells transformed with the monobody expression vector were grown in LB media to an attenuance of 0.5 at 600 nm and induced with 0.5 mM IPTG at 30 °C for 4 h. Cells were collected by centrifugation and ruptured by multiple passages through a pre-cooled microfluidizer. All the subsequent protein purification experiments were conducted at 4 °C. Cell lysate was clarified by ultracentrifugation and the soluble fraction was loaded onto Ni-NTA resin in 20 mM Tris-HCl pH 8, 500 mM NaCl and 20% glycerol. Protein was eluted using the same buffer supplemented with 500 mM imidazole, concentrated to ∼10 mg ml^−1^ and dialysed extensively against the same buffer but without imidazole.

### Protein crystallization

Crystallization experiments were all performed using the hanging-drop vapor-diffusion method at 22 °C. For DinF-BH^D40N^ crystallization, the protein samples (∼5 mg ml^−1^) were mixed with equal volume of a crystallization solution containing 100 mM HEPES-NaOH pH 7.0, 100 mM NaCl, 30–35% (v/v) PEG400, 0.02% DDM and 1 mM TCEP. Protein crystals usually appeared within 2 weeks and continued to grow to full size in a month. To obtain the ‘low pH’ crystal form, DinF-BH^D40N^ crystals were transferred into solutions containing with 100 mM Na Acetate or Citrate pH 4.0, 200 mM NaCl, 35–40% PEG400, 0.03% DDM and 1 mM TCEP, and incubated for >12 h at 22 °C. For heavy atom derivatization, protein crystals were incubated with 10 mM heavy metal compounds for 3 h at 22 °C.

For DinF-BH co-crystallization experiments, the protein (∼3 mg ml^−1^) was incubated with 0.4 mM verapamil for >24 h at 4 °C before crystallization. Then the samples were mixed with equal volume of a crystallization solution containing 100 mM Tris-HCl pH 8.0, 100 mM NaCl, 30–35% PEG400, 0.02% DDM, 1.6 mM verapamil and 1 mM TCEP. Protein crystals usually grew to full size within 2 weeks. For heavy atom derivatization, protein crystals were incubated with 5 mM heavy metal compounds for 4 h at 22 °C.

For NorM-NG crystallization, NorM-NG was first mixed with monobody at 1:1 molar ratio and then dialysed against buffer (pH ∼6) without NaCl (ref. 7). The protein sample (∼3 mg ml^−1^) was then incubated with 0.4 mM verapamil for >24 h at 4 °C before crystallization. The protein samples were mixed with equal volume of a crystallization solution containing 30–35% PEG400, 10% glycerol 0.02% DDM, 1.6 mM verapamil and 1 mM TCEP (pH ∼7). Protein crystals usually grew to full size within 5–6 weeks. For heavy atom derivatization, protein crystals were incubated with 8 mM heavy metal compounds for 4 h at 22 °C.

Notably, DinF-BH or NorM-NG was prone to precipitation if the verapamil concentration exceeded 0.5 mM under tested conditions. To enhance the ligand binding during crystallization, verapamil was added to the precipitant solutions, which markedly improved the diffraction limits of the cocrystals. Since we observed no difference between (R)- and (S)-verapamil in our functional studies (see below), we used (S)-verapamil for cocrystallization. For simplicity of discussion, we referred to (R)- and (S)-verapamil collectively as ‘verapamil’ in the text.

### Structure determination and refinement

Before data collection, crystals were plunged directly into liquid nitrogen. X-ray diffraction data were collected on the frozen crystals at 100 K. More than 5,000 crystals were screened at the beam-lines 23-ID and 22-ID in Argonne National Laboratory; only a small number (∼150) of the crystals were of data-quality. X-ray data were processed using the program suite HKL2000 (ref. 35) and further analysed using the CCP4 package^36^ unless specified otherwise. All the structures were solved by combining molecular replacement and MIRAS phasing.

The protein (DinF-BH and NorM-NG) models (PDB codes 4LZ6 and 4HUK) were placed into the unit cell by using the program PHASER^37^. Heavy-metal-binding sites were identified by difference Fourier analysis and MIRAS phases were calculated using the program SHARP^38^. Supplementary Tables 1–3 list the optimal subsets of derivative data for MIRAS phasing. The resulting electron density maps were improved by solvent flattening, histogram matching, cross-crystal averaging and phase extension^7^,^9^. Both the protruding electron densities for aromatic amino-acid side chains and heavy-metal-binding sites (Supplementary Fig. 11) were used to aid protein sequence assignment. Model building was carried out using the program O (ref. 39). Structure refinement was conducted using the program REFMAC with experimental phases as restraints^40^ to eradicate the hazards of data over-fitting (for example, narrowing the gaps between R_free_ and R_cryst_). Structural refinement statistics are shown in Supplementary Table 4.

Since DinF-BH^D40N^, verapamil-bound DinF-BH and NorM-NG were all crystallized in the same crystal forms with similar unit-cell dimensions to those of the apo- and substrate-bound DinF-BH and NorM-NG, we suggest that the differences between those structures were primarily due to the mutation, different pH (as in DinF-BH^D40N^), or verapamil binding rather than distinct crystal packing environments. We modelled (S)-verapamil in the co-structures and expect (R)-verapamil to interact with DinF-BH or NorM-NG in a similar fashion. All structure figures were prepared using the program PyMol (http://www.pymol.org).

### Drug-resistance assay

The drug export activities of DinF-BH and NorM-NG variants were evaluated based on their ability to confer cellular resistance against cytotoxic chemicals. Drug susceptibility experiments were conducted in LB medium^7^,^9^,^41^, with each assay repeated at least three times. Briefly, the exponential-phase bacterial culture from freshly transformed cells was diluted to 5 × 10^5^ colony forming units per ml with LB broth containing IPTG (0.1 mM) and ampicillin (100 μg ml^−1^) at each drug concentration. The culture was then incubated at 30 °C with shaking and the bacterial growth was monitored after ∼10 h.

Assays were performed in 96-well plates and the attenuance at 600 nm was measured using a microplate reader (Tecan GENios Plus). We defined the minimal inhibitory concentration as the lowest concentration of antimicrobial compounds that precludes growth of *E. coli* under our experimental conditions. The inhibition of drug efflux by verapamil was evaluated by its ability to potentiate the activities of various cytotoxic compounds^8^,^21^. Notably, we observed no measurable difference between (R)- and (S)-verapamil in this drug susceptibility assay.

### Drug-accumulation assay

Cultures of *E. coli* BL21 (DE3) Δ*acrAB*Δ*macAB*Δ*yojHI* cells expressing DinF-BH or NorM-NG variants were grown at 28 °C to an attenuance of ∼1.0 at 600 nm (A_600 nm_). Cells were collected, washed three times with 100 mM Tris-HCl, pH 7.0, resuspended in the same buffer to an A_600 nm_ of 1.0 ml^−1^. R6G or ethidium was added to the cells at a final concentration of 4 μg ml^−1^, followed by the addition of verapamil or carbonyl cyanide *m*-chlorophenylhydrazone (CCCP) (see below). At each time point, a 1 ml-sample was withdrawn, and cells were collected by centrifugation and washed twice with 100 mM Tris-HCl, pH 7.0. The R6G or ethidium fluorescence was then measured with the excitation and emission wavelengths of 480 or 500 nm and 570 or 580 nm, respectively^7^,^9^.

As internal controls, 25 μM CCCP was added to the cells expressing each transporter variant, and incubated at 37 °C for 15 min. CCCP, an H^+^ conductor, was used to disrupt the transmembrane proton gradient and enhance the accumulation of drugs within the cells^8^,^9^. For each MATE variant, the amount of maximum fluorescence (from the cells incubated with CCCP) was normalized to 100%. The inhibition of drug efflux by verapamil was evaluated by its ability to enhance the accumulation of R6G or ethidium within the cells, that is, to increase the R6G or ethidium fluorescence^8^. We observed no measurable difference between (R)- and (S)-verapamil in this drug-accumulation assay.

### Radioligand competition assay

[^3^H]-R6G (2.7 Ci mmol^−1^, 1.0 mCi ml^−1^) was custom-synthesized by Moravek Biochemicals (Brea, California, USA). [^3^H]-R6G binding assay was performed by using immobilized protein on Ni-NTA beads^31^,^42^ as follows. Briefly, freshly purified protein (DinF-BH or NorM-NG) was mixed gently with pre-equilibrated Ni-NTA beads at 1 mg ml^−1^ in buffers containing 20 mM Tris-HCl pH 8, 50 mM NaCl, 20% (v/v) glycerol, 0.02% (w/v) DDM, 1 mM TCEP (binding buffer) for >1 h at 4 °C. The mixture was centrifuged and the unbound material (supernatant) was discarded.

The beads were thoroughly washed, resuspended and aliquoted in the binding buffer. The aliquots were then mixed with 1 μM [^3^H]-R6G and other non-labelled ligands (R6G, ethidium or verapamil) for 1 h. The samples were then transferred into micro-columns and centrifuged; the unbound material (flow-through) was discarded. The protein was eluted by using the binding buffer supplemented with 500 mM Imidazole. The radioactivity of the eluate was measured by liquid scintillation. The amount of [^3^H]-R6G bound to the resin in the absence of protein (∼10% of the total signal) was subtracted from all measurements to account for the nonspecific binding between the Ni-NTA resin and [^3^H]-R6G. All the experiments were performed in duplicate and repeated three times.

Notably, the dissociation constant (*K*_d_) for R6G binding to DinF-BH or NorM-NG deduced from this assay (∼5 μM) was similar to that measured by using the fluorescence polarization assay^9^,^43^, thus validating this method as a ligand-binding assay for MATE transporters and further suggesting that this assay examines the ligand binding to extracellular-facing MATE transporters^10^. Furthermore, we found the inhibition constant (*K*_i_) of verapamil for DinF-BH or NorM-NG (>200 μM) to be substantially larger than that of R6G (<10 μM) or ethidium (<25 μM), suggesting weaker interactions between verapamil and the MATE transporters compared with R6G and ethidium. Of note, verapamil binds human P-glycoprotein with a K_d_ of ∼446 μM (ref. 27). We observed no measurable difference between (R)- and (S)-verapamil in this ligand-binding assay.

### Verapamil-H^+^ antiport assay

This antiport assay was conducted using everted membrane vesicles^9^,^31^ as follows. Membrane vesicles were prepared from BL21 (DE3) Δ*acrAB*Δ*macAB*Δ*yojHI* cells expressing DinF-BH variants or an empty pET-15b vector. Briefly, cells were grown in LB media to an attenuance of 0.5 at 600 nm and induced with 1 mM IPTG at 30 °C for 4 h. Cells were collected by centrifugation and washed once with buffer containing 20 mM Tris, pH 7.5, 5 mM MgCl_2_, 0.5 mM dithiothreitol and 0.25 M sucrose. The cells were then disrupted by using a pre-chilled French press and the cell lysate was centrifuged at 10,000 g for 60 min at 4 °C. Membranes were then collected by centrifugation at 100,000 g for 60 min at 4 °C.

For each measurement, membrane vesicles (50 μg total proteins) were added to 2 ml of pre-warmed (28 °C) buffer containing 20 mM Tris, pH 7.0, 5 mM MgCl_2_ and 2 μM acridine orange. The samples were continuously stirred and fluorescence was monitored with an excitation wavelength of 492 nm and emission wavelength of 525 nm using an Olis SLM-8000 spectrofluorometer. Before the addition of verapamil, 2 mM lactate was added to energize the membrane and quench acridine orange fluorescence. On the addition of 250 μM verapamil, fluorescence dequenching occurred owing to the efflux of H^+^ by antiporters that moved verapamil into the vesicles across the cell membrane. 5 mM NH_4_Cl was finally added to dissipate the H^+^ gradient. We observed no measurable difference between (R)- and (S)-verapamil in this antiport assay.

## Additional information

**How to cite this article:** Radchenko, M. *et al*. Structural basis for the blockade of MATE multidrug efflux pumps. *Nat. Commun*. 6:7995 doi: 10.1038/ncomms8995 (2015).

## Supplementary Material

Supplementary Information Supplementary Figures 1-11 and Supplementary Tables 1-4
