# Supplementary material for: Structural basis for the blockade of MATE multidrug efflux pumps
Source: Nat Commun. 2015 Aug 6;6:7995. doi: 10.1038/ncomms8995 (PMC4866600; doi:10.1038/ncomms8995)
Supplement: Supplementary Information — Supplementary Figures 1-11 and Supplementary Tables 1-4 [file ncomms8995-s1.pdf]

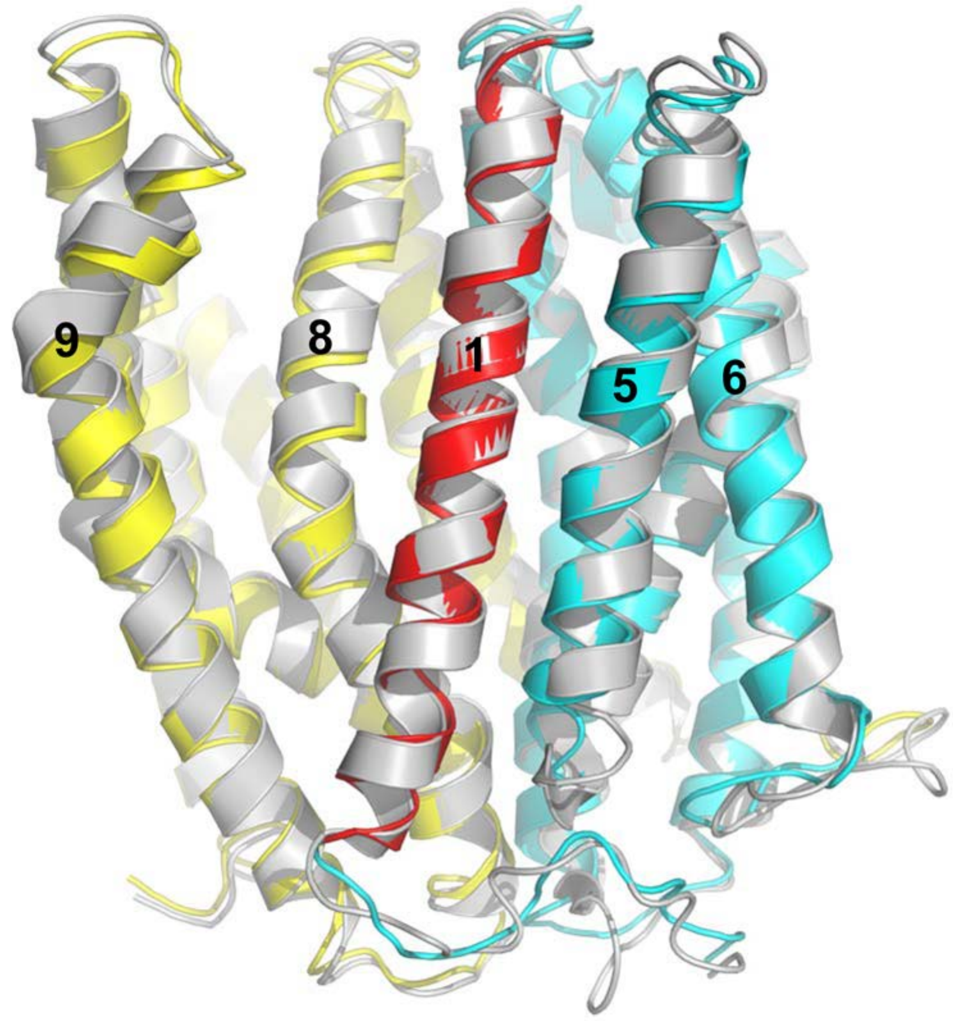

Supplementary Figure 1. Structural overlay of DinF-BH and DinF-BH^{D40N}.

The N and C domains are colored cyan and yellow respectively in DinF-BH^{D40N}, except for TM1, which is in red; the R6G-bound DinF-BH is colored in grey. Notably, TM1 is kinked in neither DinF-BH nor DinF-BH^{D40N}.

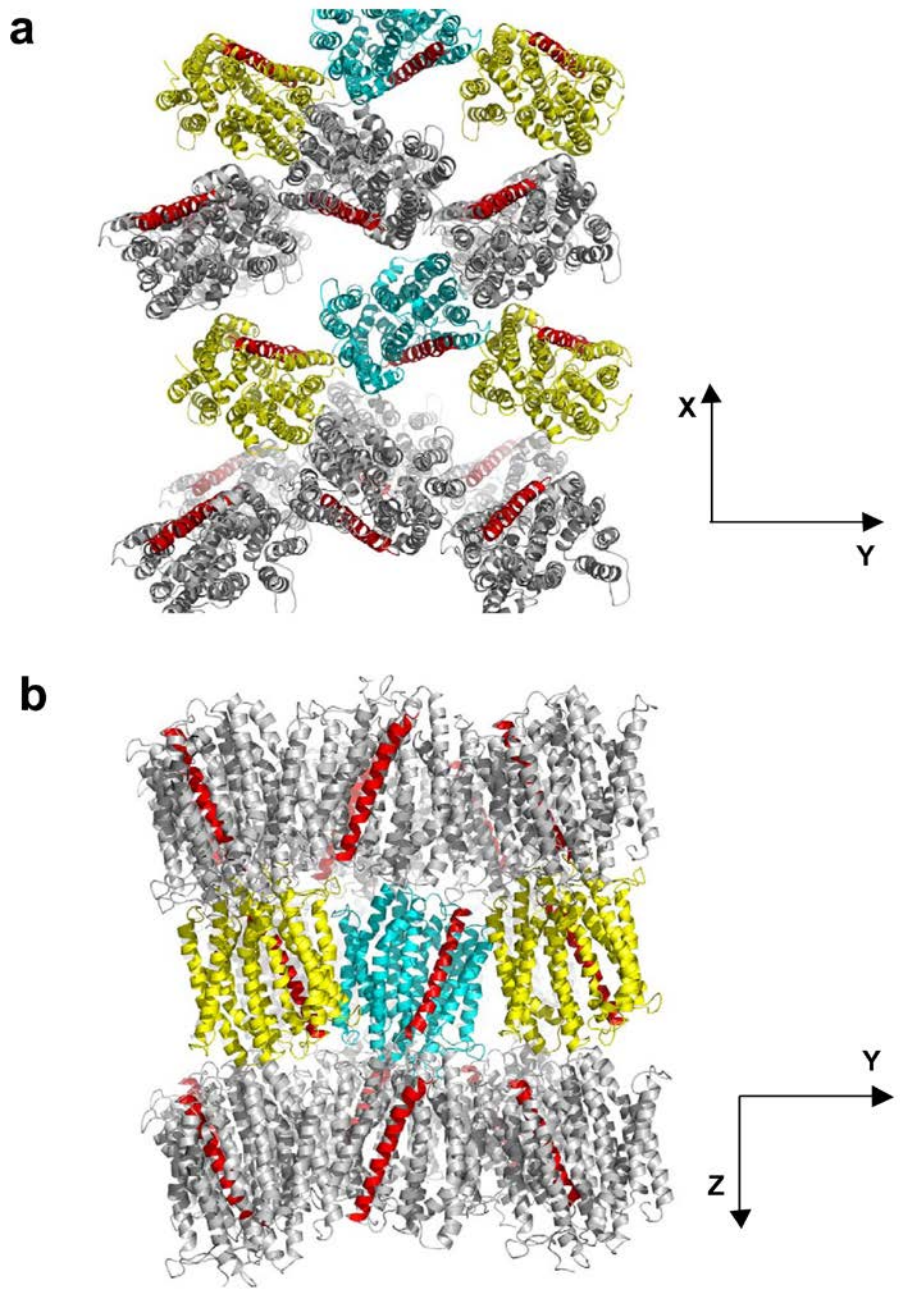

Supplementary Figure 2. Locations of TM1 within the DinF-BH^{D40N} crystals.

Protein molecules are colored cyan, yellow and grey, respectively, except for TM1, which is in red. The directions of unit cell axes are also indicated. (**a**) View of crystal packing down the Z-axis of the unit cell. It is particularly evident from this view that the TM1 is removed from any crystal packing interactions between neighboring DinF-BH^{D40N} molecules. (**b**) View down the X-axis of the unit cell.

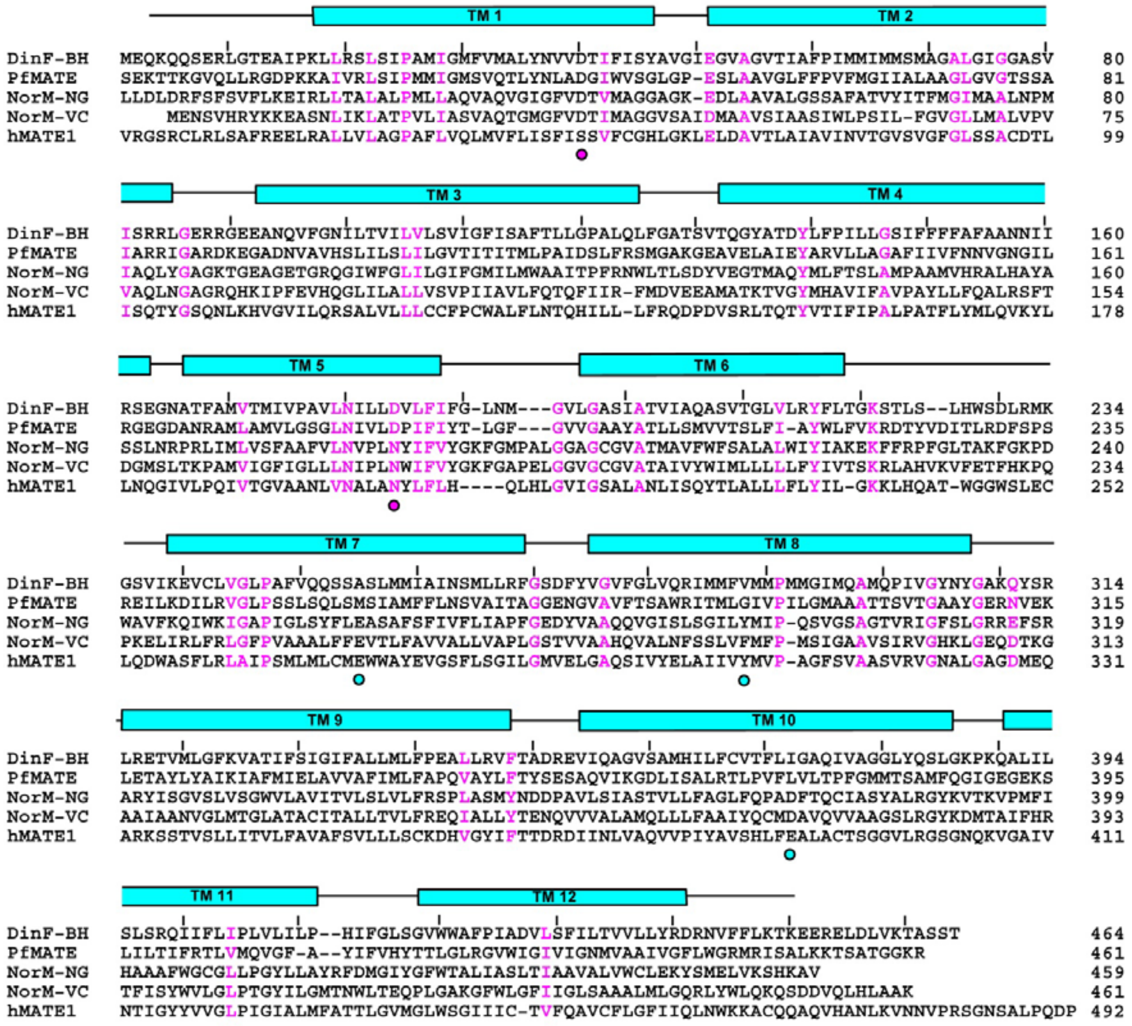

Supplementary Figure 3. Sequence alignment of representative MATE proteins.

Residues that are conserved among the five MATE proteins are colored magenta. Regions of secondary structural elements in DinF-BH are outlined, with every 10th residue marked. Magenta and cyan dots highlight amino acids that likely bind cations in DinF-BH and NorM-NG, respectively. Residues 1 in PfMATE and NorM-NG, residues 1-20 in hMATE1 were omitted for clarity. Notably, the H^+-coupled hMATE1 bears the conserved cation-binding amino acids as found in NorM-NG and NorM-VC (cyan dots), while lacking the two aspartates (magenta dots) as seen in DinF-BH and PfMATE.

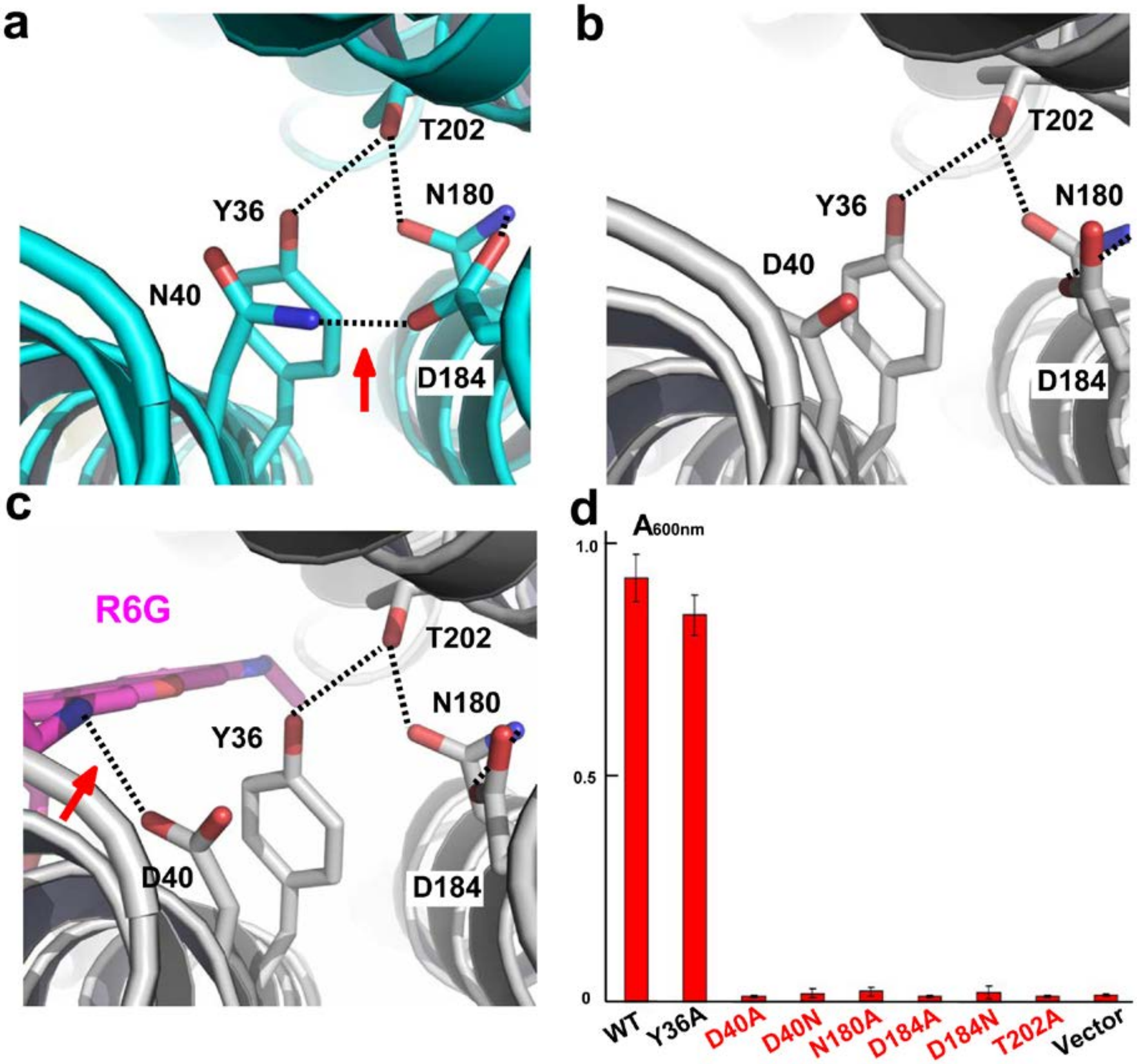
a
T202
Y36
N180
N40
D184
b
T202
Y36
N180
D40
D184
c
R6G
T202
N180
Y36
D40
D184
d
A600nm
1.0
0.5
0
WT
Y36A
D40A
D40N
N180A
D184A
D184N
T202A
Vector

Supplementary Figure 4. Structural basis for the direct competition in DinF-BH. (**a-c**) Close-up views of the H-bonding networks in DinF-BH^{D40N}(**a**), deprotonated DinF-BH (**b**), and deprotonated, R6G-bound DinF-BH (**c**). H-bonding interactions as well as the contacts between D40 and R6G were indicated by dotted-lines. The interactions between D40N and D184, and D40 and R6G were highlighted by red arrows. (**d**) The inhibition of bacterial growth as measured by attenuance at 600nm. The bacteria expressing the DinF-BH variants and pET15b were grown in the presence of 0.5 μg/ml ethidium. Error bars indicate s.d. among three biological replicates.

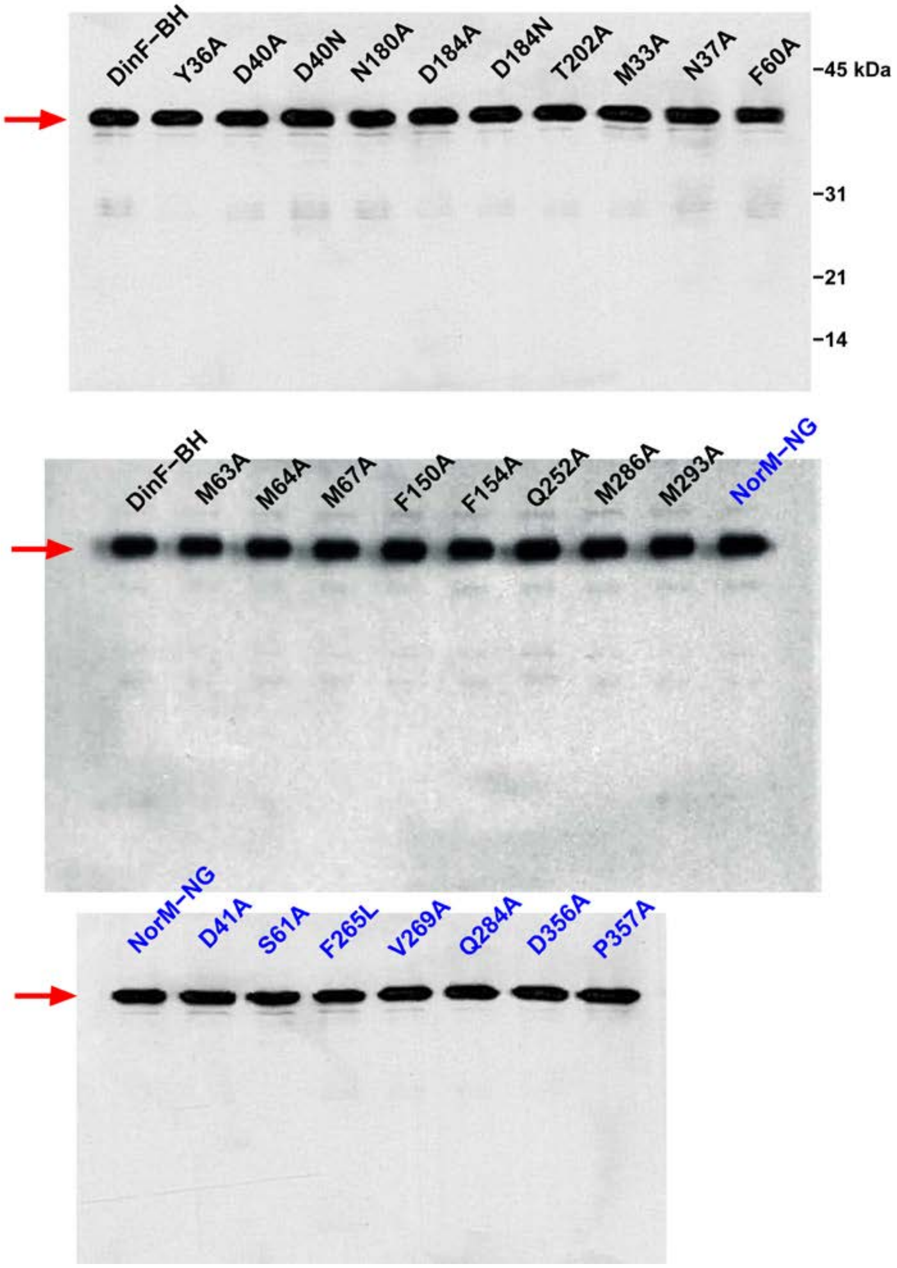

Supplementary Figure 5. Western blot analysis of MATE variants.

Western blot analysis of DinF-BH and NorM-NG variants in membrane preparations was performed by using an antibody against the His-tag. Red arrows indicate the positions of the MATE transporters. This analysis suggested that the MATE variants studied in this work were expressed at similar levels.

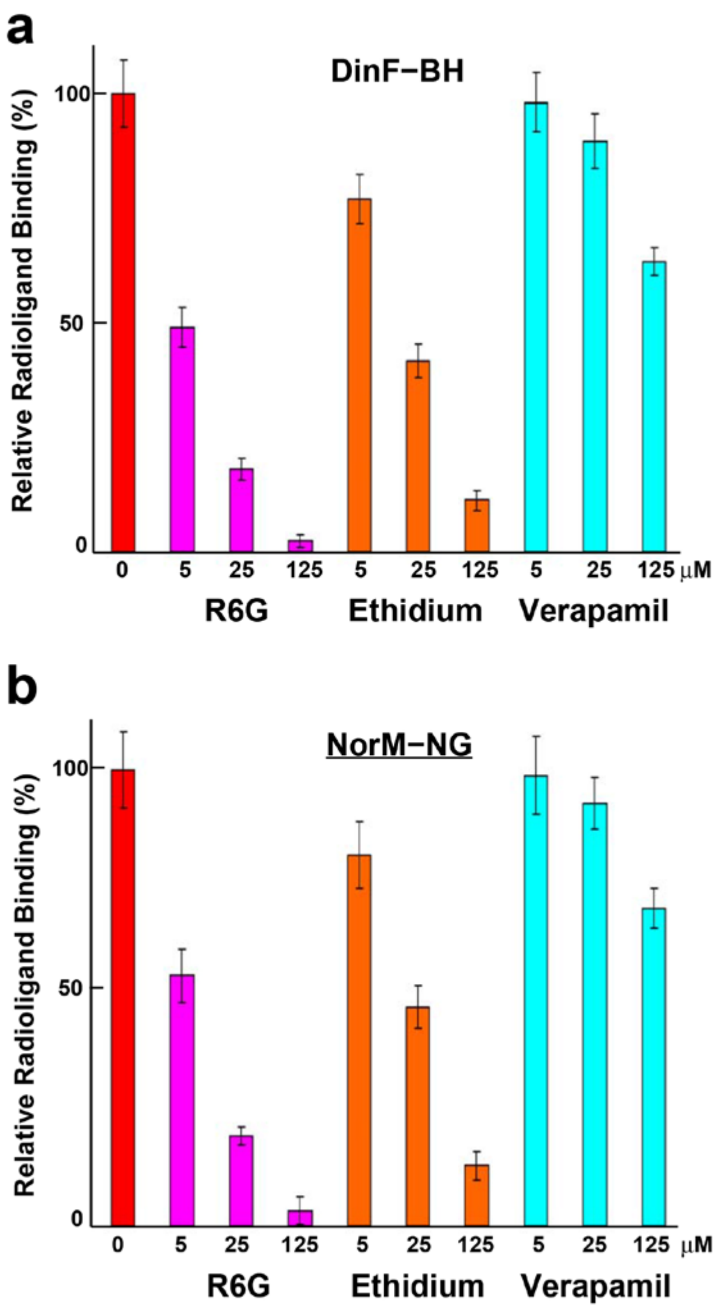

Supplementary Figure 6. Competition of radioligand binding by verapamil.

[^{3}H]-rhodamine 6G (R6G) binding by DinF-BH (**a**) and NorM-NG (**b**) and its inhibition by the addition of increasing concentrations (5, 25, or 125 μM) of non-labeled R6G (magenta column), ethidium (orange column) and verapamil (cyan column). Error bars indicate s.d. among three biological replicates.

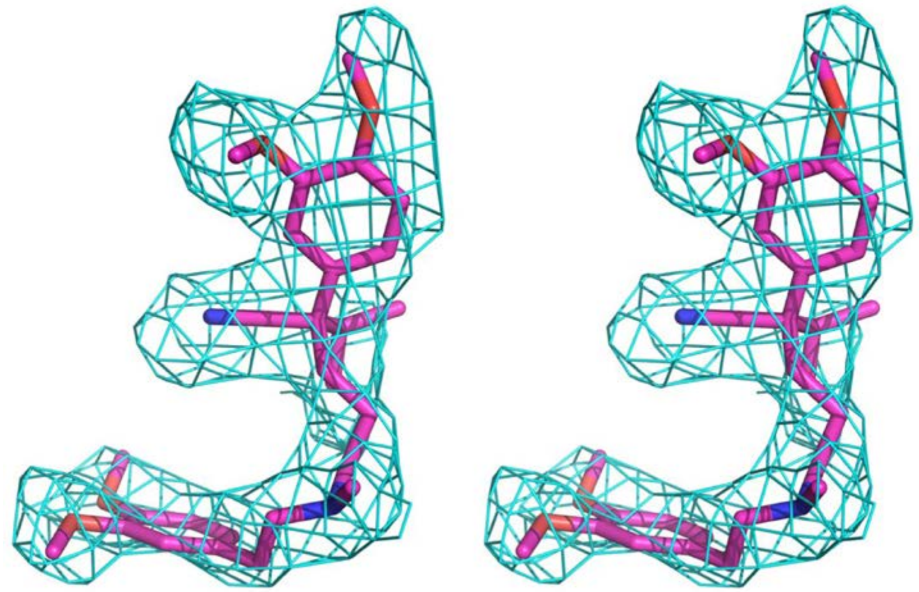

Supplementary Figure 7. Stereo view of the fitting of verapamil to the electron density in DinF-BH.

The electron density map (cyan mesh) for verapamil-bound DinF-BH was calculated to 3.0 Å resolution using native amplitudes and density-modified MIRAS phases. Density modification included solvent flattening, histogram matching, cross-crystal averaging and phase extension. The electron density was overlaid onto the final model of verapamil (magenta sticks) and contoured at 1.5 σ. Notably, the orientation of verapamil was well-defined based on the unbiased electron density.

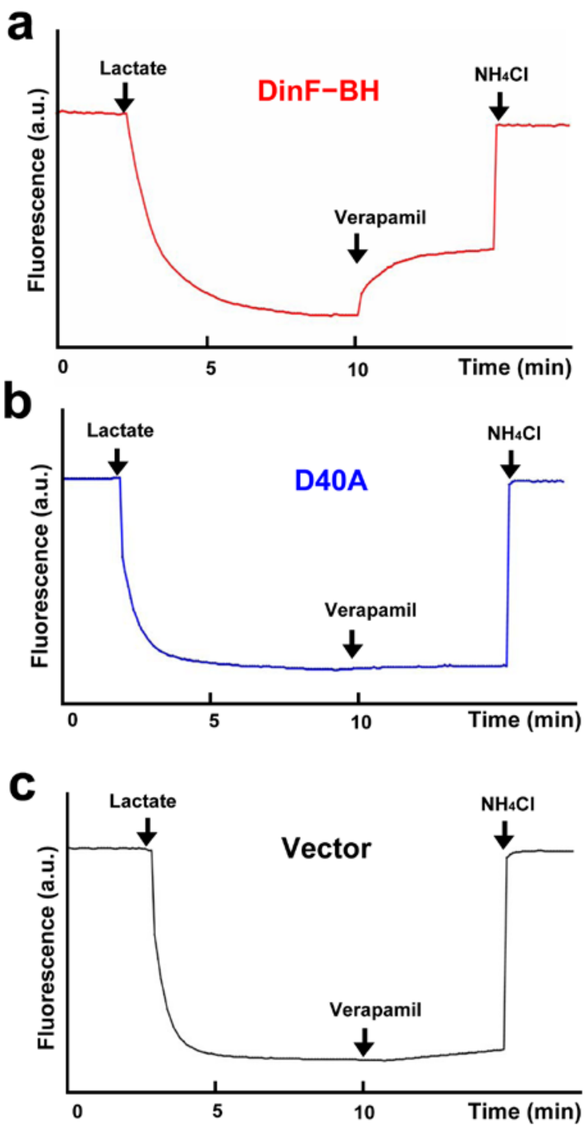

Supplementary Figure 8. Verapamil-H^+ antiport in everted membrane vesicles.

The transmembrane proton gradient was examined by measuring time-dependent, acridine orange fluorescence in everted membrane vesicles containing DinF-BH (**a**), DinF-BH^{D40A} (**b**), or pET15b (**c**). The fluorescence was shown in arbitrary units (a.u.).

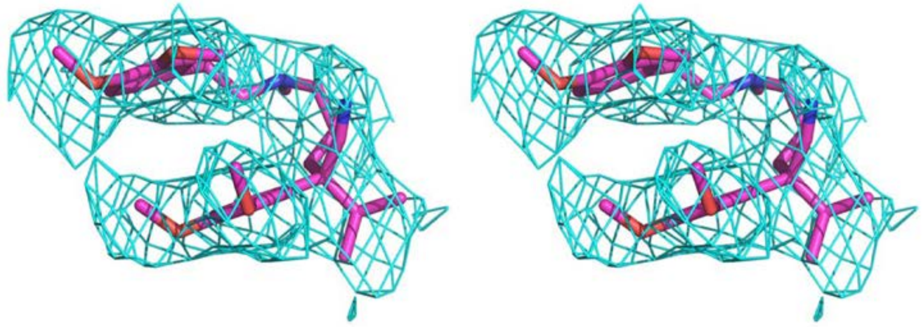

Supplementary Figure 9. Stereo view of the fitting of verapamil to the electron density in NorM-NG.

The electron density map (cyan mesh) for verapamil-bound Norm-NG was calculated to 3.0 Å resolution using density-modified MIRAS phases. The electron density was overlaid onto the final model of verapamil (magenta sticks) and contoured at 2.0 σ. The conformation of the bound verapamil was well-defined on the basis of the electron density and differs drastically from that seen in the verapamil-bound DinF-BH. Notably, the distance between the two stacked aromatic rings in verapamil is ~5 Å, and the two rings adopt a parallel displaced configuration, likely to minimize the repulsive electrostatic interactions between them.

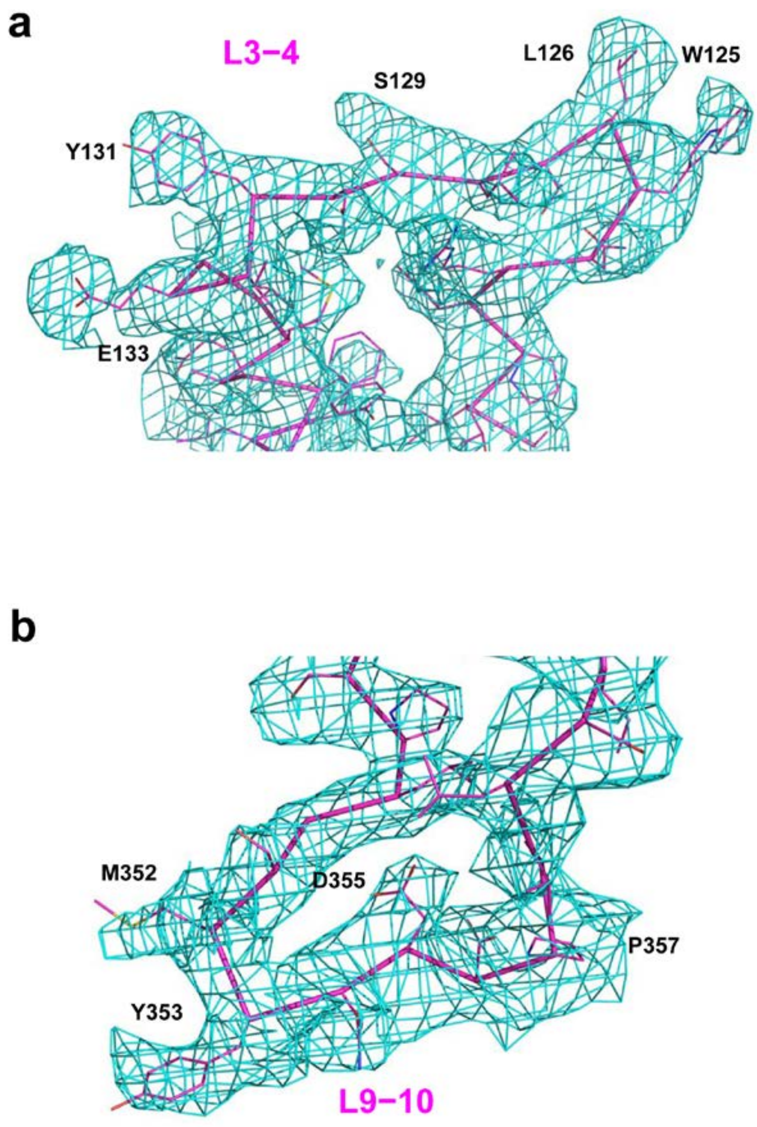

Supplementary Figure 10. Experimental electron density for verapamil-bound NorM-NG.

The featured slices of electron density depict L3-4 (**a**) and L9-10 (**b**). The electron density map (cyan mesh) was calculated to 3.0 Å resolution using density-modified MIRAS phases. The electron density was overlaid onto the final protein model (magenta sticks) and contoured at 1.5 σ. The protruding electron densities for hydrophobic side chains were useful for amino-acid sequence assignment.

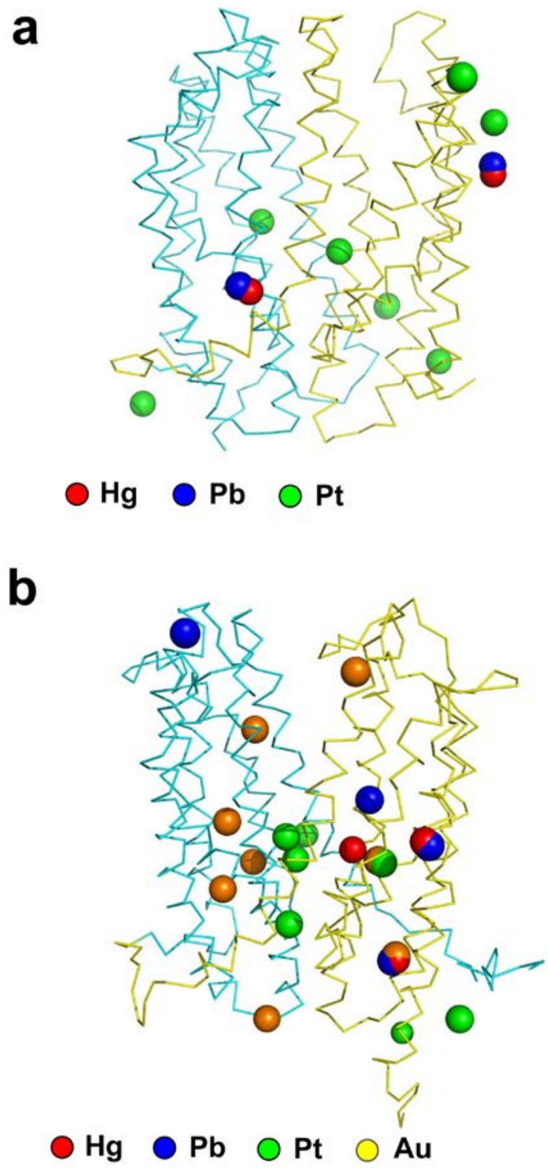

Supplementary Figure 11. Heavy-metal binding sites in DinF-BH and NorM-NG.

The Cα backbones of DinF-BH (**a**) and NorM-NG (**b**) were colored in cyan (N-terminal half) and yellow (C-terminal half). The mercury-, lead-, platinum- and gold-binding sites were highlighted by red, blue, green and orange spheres, respectively.

Supplementary Table 1. Data collection and MIRAS phasing statistics for DinF-BH^{D40N}.

	Native (low pH)	EMTS[a] (low pH)	K_2PtCl_4 (low pH)	$K_2Pt(NO_2)_4$ (low pH)	Native	EMTS	TMLA[a]	$K_2Pt(NO_2)_4$
Wavelength	1.033 Å	0.992 Å	1.042 Å	1.042 Å	1.033 Å	0.992 Å	0.932 Å	1.042 Å
Space Group	$P2_12_12_1$	$P2_12_12_1$	$P2_12_12_1$	$P2_12_12_1$	$P2_12_12_1$	$P2_12_12_1$	$P2_12_12_1$	$P2_12_12_1$
a,b,c (Å)	89.68, 93.42, 101.79	89.14, 93.30, 101.62	89.72, 93.15, 101.66	89.73, 93.25, 101.70	88.89, 92.13, 101.42	89.12, 92.65, 101.38	88.78, 92.69, 101.18	89.02, 92.20, 100.75
Resolution (Å)	100-3.00	100-4.00	100-4.50	100-4.00	100-3.50	100-4.00	100-4.00	100-4.50
Observations	137201	95068	79428	89279	217129	84725	81083	75792
Unique reflections	15805	7862	5454	7669	11572	7264	7394	5286
Completeness (last shell)	90.1% (68.5%)	98.3% (90.1%)	99.2% (92.2%)	95.9% (88.5%)	98.2% (94.7%)	90.8% (89.7%)	92.4% (83.5%)	96.1% (81.3%)
R_{sym}[b] (last shell)	7.6% (63.1%)	7.0% (67.5%)	8.6% (67.7%)	6.8% (58.6%)	4.9% (56.8%)	6.9% (58.7%)	6.5% (52.7%)	5.5% (48.7%)
I/σ (last shell)	20.2 (2.1)	21.2 (1.8)	17.2 (2.8)	19.3 (2.0)	22.6 (2.2)	24.1 (2.9)	29.9 (2.2)	26.3 (2.2)
Phasing power[c] (iso/ano)	N.A.	1.51/0.82	1.38/0.75	1.30/0.81	N.A.	1.35/0.64	1.54/0.74	1.26/0.61
R_{cullis}[d] (iso/ano)	N.A.	0.61/0.76	0.60/0.84	0.59/0.75	N.A.	0.66/0.81	0.65/0.76	0.63/0.69

Overall MIRAS figure of merit[e] (20 - 4.0 Å): 0.63 (acentric), 0.67 (centric) (low pH); 0.64 (acentric), 0.70 (centric).

[a]EMTS: thimerosal. TMLA: trimethyllead acetate.

[b]$R_{sym} = \Sigma| I - \langle I \rangle| / \Sigma I$, where I is the observed intensity of symmetry-related reflections.

[c]Phasing power= F_h / E, where F_h is the rms isomorphous/anomalous difference and E the rms residual lack-of-closure.

[d]R_{cullis}(iso)= $\Sigma(||FPH - FP| - |FH(calc)||) / \Sigma(|FPH - FP|)$, where FPH and FP are structure factors for derivative and native data, respectively. R_{cullis}(iso) is valid for centric reflections only.

[d]R_{cullis}(ano)= $\Sigma(||\Delta FPH(obs)| - |\Delta FPH(calc)||) / \Sigma|\Delta FPH(obs)|$, where ΔFPH(obs) and ΔFPH(calc) are the observed and calculated structure factor differences between Bijvoet pairs, respectively.

[e]Figure of merit is defined as weighted mean value of the cosine of phase error.

Supplementary Table 2. Data collection and phasing statistics for verapamil-bound DinF-BH.

	Native	EMTS[a]	TMLA[a]	K_2PtCl_4	$K_2Pt(NO_2)_4$
Wavelength	1.033 Å	0.992 Å	0.932 Å	1.042 Å	1.042 Å
Space Group	$P2_12_12_1$	$P2_12_12_1$	$P2_12_12_1$	$P2_12_12_1$	$P2_12_12_1$
a,b,c (Å)	91.06, 94.15, 102.46	91.38, 94.18, 102.66	91.09, 93.88, 102.48	91.07, 94.23, 102.37	91.27, 94.26, 102.51
Resolution	100-3.00 Å	100-4.00 Å	100-4.00 Å	100-4.50 Å	100-4.00 Å
Observations	235865	181720	146668	104688	220668
Unique reflections	17597	7780	7720	5259	7865
Completeness	96.2%	97.2%	96.5%	95.6%	98.3%
(last shell)	(82.3%)	(85.5%)	(88.3%)	(84.1%)	(89.9%)
R_{sym}[b]	6.4%	8.4%	7.6%	7.4%	9.3%
(last shell)	(52.8%)	(58.0%)	(52.1%)	(54.3%)	(63.1%)
I/σ	25.1	30.7	26.7	24.6	31.8
(last shell)	(2.0)	(2.2)	(2.1)	(2.0)	(2.2)
Phasing power[c] (iso/ano)	N.A.	1.58/0.96	1.48/0.97	1.27/0.86	1.32/0.82
R_{cullis}[d] (iso/ano)	N.A.	0.59/0.66	0.58/0.72	0.61/0.82	0.64/0.81
Overall MIRAS figure of merit[e] (20-4.00 Å): 0.64 (acentric), 0.66 (centric).					

[a]EMTS: thimerosal. TMLA: trimethyllead acetate.

[b]$R_{sym} = \Sigma| I - \langle I\rangle| / \Sigma I$, where I is the observed intensity of symmetry-related reflections.

[c]Phasing power= F_h / E, where F_h is the rms isomorphous/anomalous difference and E the rms residual lack-of-closure.

[d]R_{cullis}(iso)= $\Sigma(||FPH – FP| - |FH(calc)||) / \Sigma(|FPH – FP|)$, where FPH and FP are structure factors for derivative and native data, respectively. R_{cullis}(iso) is valid for centric reflections only.

[d]R_{cullis}(ano)= $\Sigma(||\Delta FPH(obs)| - |\Delta FPH(calc)||) / \Sigma|\Delta FPH(obs)|$, where ΔFPH(obs) and ΔFPH(calc) are the observed and calculated structure factor differences between Bijvoet pairs, respectively.

[e]Figure of merit is defined as weighted mean value of the cosine of phase error.

Supplementary Table 3. Data collection and phasing statistics for verapamil-bound NorM-NG.

	Native	EMTS[a]	TMLA[a]	$KAu(CN)_2$	K_2PtCl_4
Wavelength	1.033 Å	0.992 Å	0.932 Å	1.000 Å	1.042 Å
Space Group	$P3_221$	$P3_221$	$P3_221$	$P3_221$	$P3_221$
a,b,c (Å)	117.64, 117.64, 225.99	118.15, 118.15, 226.01	117.88, 117.88, 225.76	117.75, 117.75, 226.11	118.04, 118.04, 226.20
Resolution	100-3.00 Å	100-3.80 Å	100-4.00 Å	100-4.00 Å	100-4.50 Å
Observations	812647	151128	186282	204122	120388
Unique reflections	35742	17802	15488	15216	11400
Completeness	98.8%	95.2%	96.8%	95.1%	98.7%
(last shell)	(92.1%)	(85.5%)	(90.3%)	(88.1%)	(89.9%)
R_{sym}[b]	9.3%	7.4%	8.6%	8.4%	8.3%
(last shell)	(52.2%)	(51.0%)	(54.1%)	(55.3%)	(43.1%)
I/σ	16.1	26.7	27.1	22.6	21.8
(last shell)	(2.0)	(2.1)	(2.0)	(2.3)	(2.2)
Phasing power[c] (iso/ano)	N.A.	1.48/0.97	1.43/0.92	1.17/0.86	1.22/0.82
R_{cullis}[d] (iso/ano)	N.A.	0.61/0.70	0.65/0.76	0.67/0.84	0.64/0.88
Overall MIRAS figure of merit[e] (20-3.80 Å): 0.62 (acentric), 0.65 (centric).					

[a]EMTS: thimerosal. TMLA: trimethyllead acetate.

[b]$R_{sym} = \Sigma| I - \langle I \rangle| / \Sigma I$, where I is the observed intensity of symmetry-related reflections.

[c]Phasing power= F_h / E, where F_h is the rms isomorphous/anomalous difference and E the rms residual lack-of-closure.

[d]$R_{cullis}(iso)= \Sigma(||FPH – FP| - |FH(calc)||) / \Sigma(|FPH – FP|)$, where FPH and FP are structure factors for derivative and native data, respectively. $R_{cullis}(iso)$ is valid for centric reflections only.

[d]$R_{cullis}(ano)= \Sigma(||\Delta FPH(obs)| - |\Delta FPH(calc)||) / \Sigma|\Delta FPH(obs)|$, where $\Delta FPH(obs)$ and $\Delta FPH(calc)$ are the observed and calculated structure factor differences between Bijvoet pairs, respectively.

[e]Figure of merit is defined as weighted mean value of the cosine of phase error.

Supplementary Table 4. Structure refinement statistics for MATE transporters.

	$DinF\text{-}BH^{D40N}$ (low pH)	$DinF\text{-}BH^{D40N}$	Verapamil-bound DinF-BH	Verapamil-bound NorM-NG
Resolution range	20.0-3.00 Å	20.0-3.50 Å	15-3.00 Å	20.0-3.00 Å
Number of reflections	14860	10990	15934	32862
R_{cryst} [a]	28.30%	27.10%	27.11%	27.61%
R_{free} [b]	29.74%	28.54%	28.47%	29.16%
Number of atoms	3413	3413	3446	4248
$\langle B \rangle$	102.7	161.3	113.3	121.7
$\langle B \rangle_{ligand}$	N.A.	N.A.	145.2	139.2
rms deviation				
bond length,	0.012 Å,	0.010 Å,	0.012 Å,	0.012 Å,
bond angle	2.01^{o}	2.00^{o}	2.02^{o}	2.01^{o}
Ramachandran				
favored,	96.2%,	96.2%,	96.2%,	94.7%,
allowed,	3.8%,	3.8%,	3.8%,	5.3%
disallowed.	0%.	0%.	0%.	0%

[a] $R_{cryst} = \Sigma(||F_{obs}| - |F_{calc}||) / \Sigma(|F_{obs}|)$, where F_{obs} and F_{calc} are the observed and calculated structure factors, respectively.

[b] R_{free} is the same as R_{cryst} but calculated with 5% of the reflections excluded from structure refinement.
